# Using Core Genome Alignments To Assign Bacterial Species

**DOI:** 10.1128/mSystems.00236-18

**Published:** 2018-12-04

**Authors:** Matthew Chung, James B. Munro, Hervé Tettelin, Julie C. Dunning Hotopp

**Affiliations:** a Institute for Genome Sciences, University of Maryland Baltimore, Baltimore, Maryland, USA; b Department of Microbiology and Immunology, University of Maryland Baltimore, Baltimore, Maryland, USA; c Greenebaum Comprehensive Cancer Center, University of Maryland Baltimore, Baltimore, Maryland, USA; University of California, San Diego

**Keywords:** *Anaplasma*, *Rickettsia*, *Rickettsiales*, *Wolbachia*, bacterial taxonomy, core genome alignment, genomics, species concept

## Abstract

With the exponential increase in the number of bacterial taxa with genome sequence data, a new standardized method to assign species designations is needed that is consistent with classically obtained taxonomic analyses. This is particularly acute for unculturable, obligate intracellular bacteria with which classically defined methods, like DNA-DNA hybridization, cannot be used, such as those in the *Rickettsiales*. In this study, we generated nucleotide-based core genome alignments for a wide range of genera with classically defined species, as well as those within the *Rickettsiales*. We created a workflow that uses the length, sequence identity, and phylogenetic relationships inferred from core genome alignments to assign genus and species designations that recapitulate classically obtained results. Using this method, most classically defined bacterial genera have a core genome alignment that is ≥10% of the average input genome length. Both *Anaplasma* and *Neorickettsia* fail to meet this criterion, indicating that the taxonomy of these genera should be reexamined. Consistently, genomes from organisms with the same species epithet have ≥96.8% identity of their core genome alignments. Additionally, these core genome alignments can be used to generate phylogenomic trees to identify monophyletic clades that define species and neighbor-network trees to assess recombination across different taxa. By these criteria, *Wolbachia* organisms are delineated into species different from the currently used supergroup designations, while *Rickettsia* organisms are delineated into 9 distinct species, compared to the current 27 species. By using core genome alignments to assign taxonomic designations, we aim to provide a high-resolution, robust method to guide bacterial nomenclature that is aligned with classically obtained results.

**IMPORTANCE** With the increasing availability of genome sequences, we sought to develop and apply a robust, portable, and high-resolution method for the assignment of genera and species designations that can recapitulate classically defined taxonomic designations. Using cutoffs derived from the lengths and sequence identities of core genome alignments along with phylogenetic analyses, we sought to evaluate or reevaluate genus- and species-level designations for diverse taxa, with an emphasis on the order *Rickettsiales*, where species designations have been applied inconsistently. Our results indicate that the *Rickettsia* genus has an overabundance of species designations, that the current *Anaplasma* and *Neorickettsia* genus designations are both too broad and need to be divided, and that there are clear demarcations of *Wolbachia* species that do not align precisely with the existing supergroup designations.

## Abstract (precis)

With the increasing availability of genome sequences, we sought to develop and apply a robust, portable, and high-resolution method for the assignment of genera and species designations that can recapitulate classically defined taxonomic designations. Using cutoffs derived from the lengths and sequence identities of core genome alignments along with phylogenetic analyses, we sought to evaluate or reevaluate genus- and species-level designations for diverse taxa, with an emphasis on the order *Rickettsiales*, where species designations have been applied inconsistently. Our results indicate that the *Rickettsia* genus has an overabundance of species designations, that the current *Anaplasma* and *Neorickettsia* genus designations are both too broad and need to be divided, and that there are clear demarcations of *Wolbachia* species that do not align precisely with the existing supergroup designations.

## INTRODUCTION

While acknowledging the disdain that some scientists have for taxonomy, Stephen Jay Gould frequently highlighted in his writings how classifications arising from a good taxonomy both reflect and direct our thinking, stating, “the way we order reflects the way we think. Historical changes in classification are the fossilized indicators of conceptual revolutions” (1). Historically, bacterial species delimitation relied on phenotypic, morphologic, and chemotaxonomic characterizations (2–4). The 1960s saw the introduction of molecular techniques in bacterial species delimitation through the use of GC content (5), DNA-DNA hybridization (DDH) (6), and 16S rRNA sequencing (7, 8). Currently, databases like SILVA (9) and Greengenes (10) use 16S rRNA sequencing to identify bacteria. However, 16S rRNA sequencing often fails to separate closely related taxa, and its utility for species-level identification is questionable (10–12). Multilocus sequence analysis (MLSA) has also been used to delineate species (13), as has the phylogenetic analysis of both rRNA and protein-coding genes (3, 14, 15). Nongenomic mass spectrometry-based approaches, in which expressed proteins and peptides are characterized, provide complementary data to phenotypic and genomic species delimitations (16, 17) and are used in clinical microbiology laboratories. However, DDH remains the “gold standard” of defining bacterial species (18, 19), despite the intensive labor involved and its inability to be applied to nonculturable organisms. A new genome-based bacterial species definition is attractive, given the increasing availability of bacterial genomes, rapid sequencing improvements with decreasing sequencing costs, and data standards and databases that enable data sharing.

Average nucleotide identity (ANI) and digital DDH (dDDH) were developed as genomic-era tools that allow for bacterial species classification with a high correlation to results obtained using wet lab DDH, while bypassing the associated difficulties (20–22). For ANI calculations, the genome of the query organism is split into 1-kbp fragments, which are then searched against the whole genome of a reference organism. The average sequence identity of all matches having >60% overall sequence identity over >70% of their length is defined as the ANI between the two organisms (18). dDDH uses the sequence similarity of conserved regions between two genomes of interest (23) to calculate genome-to-genome distances. These distances are converted to a dDDH value, which is intended to be analogous to DDH values obtained using traditional laboratory methods (23). There are three formulas for calculating dDDH values between two genomes using either (i) the length of all matches divided by the total genome length, (ii) the sum of all identities found in matches divided by the overall match length, and (iii) the sum of all identities found in matches divided by the total genome length, with the second formula being recommended for assigning species designations for draft genomes (24, 25). However, neither ANI nor dDDH reports the total length of fragments that match the reference genome, and problems arise when only a small number of fragments are unknowingly used. Additionally, neither method generates an alignment that can be used for complementary phylogenetic analyses, instead relying solely on strict cutoffs to define species, potentially leading to the formation of paraphyletic taxa.

Some recent phylogenomic analyses have shifted toward using the core proteome, a concatenated alignment constructed using the amino acid sequences of genes shared between the organisms of interest (26). However, differences in annotation that affect gene calls can add an unnecessary variable when deriving evolutionary relationships. Instead, we propose that such analyses should use nucleotide core genome alignments to infer phylogenetic relationships with a well-defined nucleotide identity threshold to identify where trees should be pruned to define species. Such an analysis would be enabled by genome aligners like Mauve (27) and Mugsy (28), which identify regions of nucleotide identity that are shared between genomes, which are referred to as locally collinear blocks (LCBs). For any given subset of genomes, a core genome alignment can be generated by concatenating these LCBs and retaining only positions present in all genomes. Using the length and sequence identity of the core genome alignment, paired with a phylogeny generated from this alignment, genus- and species-level taxonomic assignments can be developed. A core genome alignment-based method provides advantages to its protein-based counterpart in that it is of a higher resolution and independent of annotation, while also being transparent with respect to the data used in the calculations and very amenable to data sharing and deposition in data repositories.

The *Rickettsiales* are an order within the *Alphaproteobacteria* composed of obligate, intracellular bacteria where classic DNA-DNA hybridization is not possible and bacterial taxonomy is uneven, with each of the genera having its own criteria for assigning genus and species designations. Within the *Rickettsiales*, there are three major families, the *Anaplasmataceae*, *Midichloriaceae*, and *Rickettsiaceae*, with an abundance of genomic data being available for genera within the *Anaplasmataceae* and *Rickettsiaceae.* However, as noted above, species definitions in these families are inconsistent, and organisms in the *Wolbachia* genus lack community-supported species designations. While the *Wolbachia* community has vigorously discussed nomenclature at each of its biannual meetings over the past 20 years, thus far, the community has supported only supergroup designations. Proposed ANI- and dDDH-informed *Wolbachia* species definitions and nomenclature (29) lack support from the *Wolbachia* community for a number of reasons (see, e.g., reference 30).

The last reorganization of the *Rickettsiaceae* taxonomy occurred in 2001 (31), a time when there were <300 sequenced bacterial genomes (32), a limited number of *Rickettsia* genomes (33, 34), and no *Anaplasmataceae* genomes (35–38). As of 2014, there were >14,000 bacterial genomes sequenced, and with this increase in available genomic information, more-informed decisions can be made with regard to taxonomic classification (32). By using core genome alignments, we can condense whole genomes into positions shared between the input genomes and use sequence identity to infer phylogenomic relationships. We can identify sequence identity thresholds for these alignments that are consistent with classically defined bacterial species and can be overlaid on phylogenetic trees generated from the same alignment. This combined approach, which we present in this study, enables a phylogenetically informed whole-genome approach to bacterial taxonomy. We apply this approach to organisms in the *Rickettsiales*, including *Rickettsia*, *Orientia*, *Ehrlichia*, *Neoehrlichia*, *Anaplasma*, *Neorickettsia*, and *Wolbachia*.

## RESULTS

### Advantages of nucleotide alignments over protein alignments for bacterial species analyses.

While core protein alignments are increasingly used for phylogenetics (39, 40), a core nucleotide alignment has more phylogenetically informative positions in the absence of substitution saturation (41), yielding a greater potential for phylogenetic signal. Nucleotide-based analyses outperform amino acid-based analyses at all time scales in terms of resolution, branch support, and congruence with independent evidence (42–44). Nucleotide and protein core genome alignments were constructed for 10 complete *Wolbachia* genomes (see Table S1 in the supplemental material), and the resulting phylogenies were compared.

10.1128/mSystems.00236-18.7 Table S1 Ten complete *Wolbachia* genomes used for PhyloPhlAn and nucleotide and protein core genome analyses. Download Table S1, XLSX file, 0.01 MB. Copyright © 2018 Chung et al. 2018 Chung et al. This content is distributed under the terms of the Creative Commons Attribution 4.0 International license.

There are a number of whole-genome aligners that can be used to generate nucleotide core genome alignments (for an overview, see reference 45). We generated core genome alignments for the 10 *Wolbachia* genomes using Mauve and Mugsy, currently two of the most commonly used whole-genome aligners (27, 28). Despite the different algorithms used by each of the two programs, the lengths of the generated core genome alignments are similar, with the Mauve and Mugsy core genome alignments being 682,949 and 579,495 bp in length, respectively. However, because Mugsy has been found to be better at handling larger genome data sets (28), Mugsy was used for all subsequent core genome alignment analyses discussed. Despite the presence of large-scale genome rearrangements present between the 10 *Wolbachia* genomes (Fig. S1), the resulting alignment is 45.9% of the average input genome length, indicating that collinear genomes are not necessarily required to construct core genome alignments. The lack of synteny results merely in more and smaller local colinear blocks.

10.1128/mSystems.00236-18.1FIG S1Synteny between the 10 complete *Wolbachia* genomes. Syntenies between the 10 complete *Wolbachia* genomes were compared and visualized using the Artemis Comparative Tool. The red ribbons indicate conserved regions of >3 kbp between two genomes, while the blue ribbons indicate >3-kbp inverted conserved regions. *Wolbachia* supergroups A (●), B (▲), C (■), D (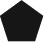), E (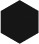) and F (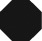) are represented in the genome subset. Shapes of the same color indicate that the multiple genomes are of the same species as determined using our determined CGASI cutoff of ≥96.8%. Download FIG S1, TIF file, 2.5 MB.Copyright © 2018 Chung et al.2018Chung et al.This content is distributed under the terms of the Creative Commons Attribution 4.0 International license.

**FIG 1:**
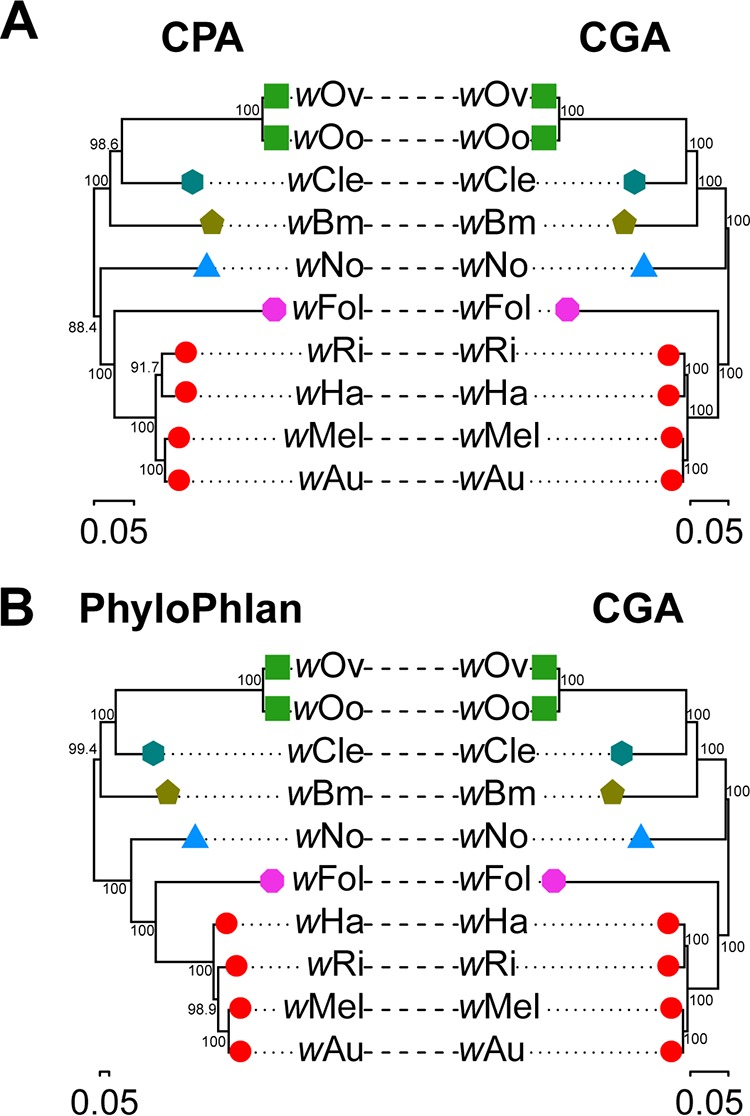
Comparison of phylogenomic trees generated using the core nucleotide alignments versus protein-based alignments for 10 complete *Wolbachia* genomes. A maximum-likelihood phylogenomic tree generated from the core genome alignment (CGA) of 10 complete *Wolbachia* genomes was compared to a core protein alignment (CPA) containing 152 genes present in only one copy (A) and an alignment generated using PhyloPhlAn, containing 176 conserved proteins (B). *Wolbachia* supergroups A (●), B (▲), C (■), D (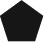), E (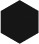), and F (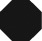) are represented in the genome subset. Shapes of the same color indicate that the multiple genomes are of the same species as defined using our determined CGASI cutoff of ≥96.8%. When comparing the ML trees generated using the core nucleotide and core protein alignments, the trees are largely similar in both topology and branch length. In the comparison between the ML trees generated using the core nucleotide alignment and PhyloPhlAn, the trees are similar except for the relationship of *w*Ri, which is sister to *w*Ha in the core nucleotide alignment tree and sister to *w*Au + *w*Mel in the PhyloPhlAn tree. Despite differences in clustering, the core nucleotide alignment ML tree consistently has higher bootstrap values than its core protein alignment or PhyloPhlAn counterpart.

Using the same 10 *Wolbachia* genomes and the available annotation at NCBI RefSeq, a core protein alignment was generated using FastOrtho, a reimplementation of the tool OrthoMCL (78). Despite the presence of different annotation methods used for gene calls, we were able to generate a protein alignment of 77,868 positions from the concatenated alignments of 152 shared genes between the 10 genomes using the available protein amino acid sequences (Table S1). In total, the core protein alignment contained 16,241 parsimony-informative positions compared to the 124,074 such positions observed with the core nucleotide alignment, indicating a 10-fold increase in potentially informative positions (Fig. 1A). When maximum-likelihood (ML) trees from the core protein alignment and the core nucleotide alignment are compared, the two trees are quite similar in topology and branch length. However, the nodes on the ML tree generated using the core nucleotide alignment consistently have higher bootstrap support values.

Similarly, we compared the *Wolbachia* core nucleotide alignment to a protein-based alignment generated using PhyloPhlAn (39). PhyloPhlAn uses a set of 400 of the most conserved proteins across diverse bacterial taxa to infer phylogenetic relationships. Across the 10 complete *Wolbachia* genomes, 176 of these 400 genes were identified to be present in each of the genomes. A concatenated amino acid alignment of these 176 genes yields an alignment with 2,877 positions, of which 2,447 are parsimony informative, indicating a 50-fold decrease in the number of parsimony-informative positions from that of the core nucleotide alignment and an 8-fold decrease relative to that of the core protein alignment. As with the core protein alignment, the ML trees constructed using the *Wolbachia* core nucleotide alignment and PhyloPhlAn have similar topologies and relative branch lengths, with the core nucleotide alignment having higher support values on its nodes (Fig. 1B). The one difference in clustering occurs within the supergroup A *Wolbachia*, in which *w*Ri clusters with *w*Ha in the core nucleotide alignment, while *w*Ri clusters with *w*Mel and *w*Au in the PhyloPhlAn ML tree. The *w*Ri and *w*Ha branch is supported by a bootstrap value of 100 in the core nucleotide alignment ML tree, while the *w*Ri, *w*Mel, and *w*Au branch is supported by a bootstrap value of 98.9 in the PhyloPhlAn ML tree.

Collectively, these results demonstrate that while nucleotide and protein alignments give roughly the same result, there are key differences. Core nucleotide alignments have almost an order of magnitude more parsimony-informative positions, and this results in stronger branch support values in the corresponding phylogenies.

### Can core genome alignments be constructed from bacteria within a genus?

To compare differences between existing classically defined species designations by this method of using sequence identity thresholds with whole-genome phylogenies, core genome alignments were constructed for seven bacterial genera representative of six different bacterial taxonomic classes (Table 1). For these seven genera, which include *Arcobacter*, *Caulobacter*, *Erwinia*, *Neisseria*, *Polaribacter*, *Ralstonia*, and *Thermus*, each of the generated core genome alignments were found to be ≥0.33 Mbp in size, representative of ≥13.6% of the average input genome size (Table 1; Table S2). For this diverse group of genera, the core genome alignments comprise a large portion of their input genomes, indicating that this technique is applicable to a wide range of classically defined bacterial taxa. Additionally, core genome alignments were successfully constructed from the genomes of four of the six *Rickettsiales* genera, including 69 *Rickettsia* genomes, 3 *Orientia* genomes, 16 *Ehrlichia* genomes, and 23 *Wolbachia* genomes (Table 1). Each of these four core genome alignments are >0.18 Mbp in length and contain >10% of the average size of the input genomes.

**TABLE 1:** Core genome alignment statistics

Genus or genus and species	Family	Class	No. of genomes analyzed	No. of established species represented	Avg genome size (Mbp)	Core genome alignment size (Mbp)	% core genome composition	No. of LCBs identified	Minimum CGASI (%)
*Rickettsia*	*Rickettsiaceae*	*Alphaproteobacteria*	69	27	1.32	0.56	42.40	36,667	81.70
*Orientia*	*Rickettsiaceae*	*Alphaproteobacteria*	3	1	2.04	0.97	47.50	12,101	96.30
*Ehrlichia*	*Anaplasmataceae*	*Alphaproteobacteria*	16	4	1.23	0.49	39.80	1,423	81.60
*Neoehrlichia*	*Anaplasmataceae*	*Alphaproteobacteria*	1	1	1.27				
*Neoehrlichia* with *Ehrlichia*	*Anaplasmataceae*	*Alphaproteobacteria*	17	5	1.24	0.11	8.90	1,661	80.10
*Anaplasma*	*Anaplasmataceae*	*Alphaproteobacteria*	30	3	1.39	0.02	1.40	33,010	84.00
A. phagocytophilum	*Anaplasmataceae*	*Alphaproteobacteria*	20	1	1.5	1.25	83.30	31,062	98.90
A. marginale or A. centrale	*Anaplasmataceae*	*Alphaproteobacteria*	10	2	1.18	0.77	65.30	2,339	90.60
*Neorickettsia*	*Anaplasmataceae*	*Alphaproteobacteria*	4	3	0.87	0.02	2.30	55	85.70
*Neorickettsia* excluding *N. helminthoeca* Oregon	*Anaplasmataceae*	*Alphaproteobacteria*	3	2	0.87	0.76	87.40	31	85.70
*Wolbachia*	*Anaplasmataceae*	*Alphaproteobacteria*	23		1.22	0.18	14.80	14,193	77.20
*Wolbachia* excluding *w*Ppe	*Anaplasmataceae*	*Alphaproteobacteria*	22		1.23	0.54	43.90	14,146	80.10
*Arcobacter*	*Campylobacteraceae*	*Epsilonproteobacteria*	44	12	2.27	0.43	18.90	34,089	78.90
*Caulobacter*	*Caulobacteraceae*	*Alphaproteobacteria*	26	4	4.97	1.61	32.40	80,702	82.50
*Erwinia*	*Enterobacteriaceae*	*Gammaproteobacteria*	22	10	4.42	0.6	13.60	17,290	78.80
*Neisseria*	*Neisseriaceae*	*Betaproteobacteria*	66	10	2.24	0.33	14.70	64,633	80.50
*Polaribacter*	*Flavobacteriaceae*	*Flavobacteriia*	24	11	3.55	0.8	22.50	20,905	77.90
*Ralstonia*	*Burkholderiaceae*	*Betaproteobacteria*	21	3	5.44	1.24	22.80	27,112	82.20
*Thermus*	*Thermaceae*	*Deinococci*	19	11	2.29	1.1	48.00	13,251	80.90

10.1128/mSystems.00236-18.8 Table S2 Genomes used for ANI, dDDH, and CGASI analysis. Download Table S2, TXT file, 0.5 MB. Copyright © 2018 Chung et al. 2018 Chung et al. This content is distributed under the terms of the Creative Commons Attribution 4.0 International license.

### Substitution saturation.

A common caveat in nucleotide alignments is the presence of substitution saturation, an issue in which the phylogenetic distances reported by nucleotide alignments are underestimated due to an overabundance of reverse mutations within the organism subset (41). Compared to protein-based methods, substitution saturation more heavily impacts nucleotide-based phylogenetic distance measurements due to the higher rate of substitutions per position. However, for each core genome alignment, when the uncorrected pairwise genetic distances are plotted against the model-corrected distances, linear relationships are observed for all alignments (*r^2^* > 0.995), indicating that substitution saturation does not hinder the ability of the core genome alignments to represent evolutionary relationships (46) (Fig. S2). Given the fact that the analyzed genera span bacterial taxonomic classes, including *Alphaproteobacteria*, *Betaproteobacteria*, *Gammaproteobacteria*, *Epsilonproteobacteria*, *Flavobacteriia*, and *Deinococci* (Table 1), we expect this result to be broadly applicable to bacterial core genome alignments.

10.1128/mSystems.00236-18.2 FIG S2 Assessing substitution saturation for core genome alignments. For each of the 14 core genome alignments that comprise ≥10% of the average input genome size, the uncorrected genetic distance between each of the members was plotted against the Tn*69*-model corrected genetic distance. The red line represents the best-fit line for each data set, while the black dotted line represents the identity line (*y* = *x*). In all cases, the relationship between the two distances are linear (*r*^2^ > 0.995), indicating little substitution saturation in the core genome alignments. Download FIG S2, TIF file, 1.6 MB. Copyright © 2018 Chung et al. 2018 Chung et al. This content is distributed under the terms of the Creative Commons Attribution 4.0 International license.

### Assessment of genus designations.

We found our initial core genome alignments for *Anaplasma* and *Neorickettsia* to be considerably shorter than other core genome alignments, both being ∼20 kbp and accounting for ≤2.3% of the average input genome sizes. The small size of the core genome alignment indicates that the *Anaplasma* and *Neorickettsia* genome subsets each contain a diverse set of genomes that are likely not of the same genus. Using input genomes from different genera to construct a core genome alignment yields an alignment of an insufficient size to accurately represent the evolutionary distances between the input genomes. For example, when the genome of *Neoehrlichia lotoris* is supplemented with the genomes used to create the 0.49-Mbp *Ehrlichia* core genome alignment, the resultant core genome alignment is 0.11 Mbp, representing only 8.9% of the average input genome size, compared to the prior 39.8%. Therefore, we used subsets of species to test whether the *Anaplasma* and *Neorickettsia* genera are too broadly defined. A core genome alignment generated using only the 20 A. phagocytophilum genomes in the 30 *Anaplasma* genome set is 1.25 Mbp and represents 83.3% of the average input genome size, while a core genome alignment of the remaining 10 *Anaplasma* genomes is 0.77 Mbp, 65.3% of the average genome input size. This result suggests that the *Anaplasma* genus should be split into two separate genera. Similarly, when the genome of *Neorickettsia helminthoeca* Oregon is excluded from the *Neorickettsia* core genome alignment, a 0.77-Mbp *Neorickettsia* core genome alignment is generated, 87.4% of the average input genome size, suggesting that *N. helminthoeca* Oregon is not of the same genus as the other three *Neorickettsia* genomes. For the remainder of this paper, these genus reclassifications are used. Given these collective results, we recommend that the genus classification level can be defined as a group of genomes that together will yield a core genome alignment that represents ≥10% of the average input genome sizes.

### Identifying a CGASI for species delineation.

**FIG 2:**
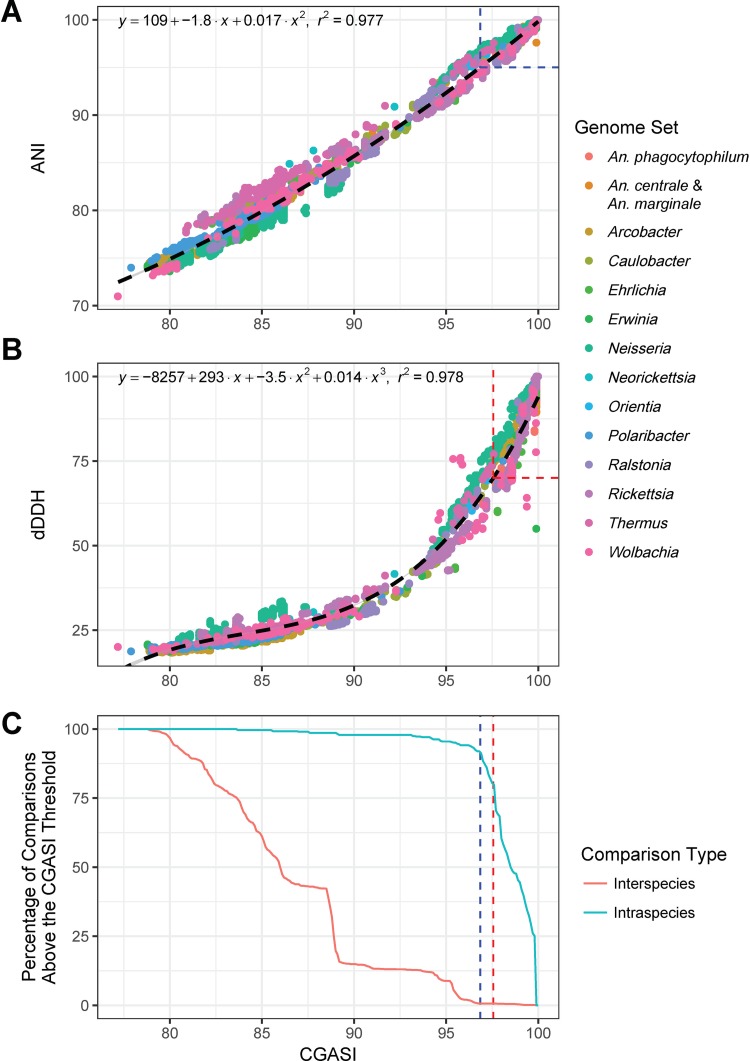
ANI, dDDH, and CGASI correlation analysis. CGASI, ANI, and dDDH values were calculated for 7,264 intragenus pairwise comparisons of genomes for *Rickettsia*, *Orientia*, *Ehrlichia*, *Anaplasma*, *Neorickettsia*, *Wolbachia*, *Caulobacter*, *Erwinia*, *Neisseria*, *Polaribacter*, *Ralstonia*, and *Thermus*. (A) The CGASI and ANI values for the intragenus comparisons follow a second-degree polynomial model (*r^2^* = 0.977), with the ANI species cutoff of ≥95% being equivalent to a CGASI of 96.8%, indicated by the blue dashed box. (B) The CGASI and dDDH values for all pairwise comparisons follow a third-degree polynomial model (*r^2^* = 0.978), with the dDDH species cutoff of ≥70% being equivalent to a CGASI of 97.6%, indicated by the red dashed box. (C) To identify the optimal CGASI cutoff to use when classifying species, for each increment of the CGASI species cutoff plotted on the *x* axis, the percentage of intraspecies and interspecies comparisons correctly assigned was determined based on classically defined species designations. The ideal cutoff should maximize the prediction of classically defined species for both interspecies and intraspecies comparisons. The ANI-equivalent CGASI species cutoff is represented by the blue dashed line, while the dDDH-equivalent CGASI species cutoff is represented by the red dashed line.

Within the *Rickettsia*, *Orientia*, *Ehrlichia*, *Anaplasma*, *Neorickettsia*, *Wolbachia*, *Arcobacter*, *Caulobacter*, *Erwinia*, *Neisseria*, *Polaribacter*, *Ralstonia*, and *Thermus* genome subsets, ANI, dDDH, and core genome alignment sequence identity (CGASI) values were calculated for 7,264 intragenus pairwise genome comparisons (Table S3), of which 601 are between members of the same species. The ANI and CGASI follow a second-degree polynomial relationship (*r^2^* = 0.977) (Fig. 2A). Using this model, the ANI species cutoff of ≥95% is analogous to a CGASI cutoff of ≥96.8%. dDDH and CGASI follow a third-degree polynomial model (*r^2^* = 0.978), with a dDDH of 70% being equivalent to a CGASI of 97.6%, indicating that the dDDH species cutoff is generally more stringent than the ANI species cutoff (Fig. 2B).

10.1128/mSystems.00236-18.9 Table S3 ANI, dDDH, and CGASI values for 7,264 interspecies comparisons of the genera *Rickettsia*, *Orientia*, *Ehrlichia*, *Anaplasma*, *Neorickettsia*, *Wolbachia*, *Arcobacter*, *Caulobacter*, *Erwinia*, *Neisseria*, *Polaribacter*, *Ralstonia*, and *Thermus*. Download Table S3, TXT file, 0.01 MB. Copyright © 2018 Chung et al. 2018 Chung et al. This content is distributed under the terms of the Creative Commons Attribution 4.0 International license.

The ideal CGASI threshold for species delineation would maximize the prediction of classically defined species, neither creating nor destroying the majority of the classically defined species. To examine this, all 7,264 intragenus pairwise comparisons were classified as either intraspecies or interspecies. Intraspecies comparisons are comparisons between genomes with the same classically defined species designation, while interspecies comparisons are between genomes within the same genus but with different classically defined species designations. Every possible CGASI threshold value was then tested for the ability to recapitulate these classically defined taxonomic classifications (Fig. 2C). In all cases, an abnormally high number of interspecies *Rickettsia* comparisons were found above both the established ANI and the dDDH species thresholds, consistent with previous observations that guidelines for establishing novel *Rickettsia* species are too lax (47), and as such they were excluded from this specific analysis. Below a CGASI of 97%, classically defined species begin to be separated, while organisms classically defined as different species begin to be collapsed. This coincides with the above-calculated ANI-equivalent threshold but differs from the above-calculated dDDH equivalent (Fig. 2C). The dDDH-equivalent CGASI threshold of 97.6% failed to predict the classically defined taxa from 100 intraspecies comparisons (Table S3) while the ANI-equivalent CGASI threshold of 96.8% failed to predict the classically defined taxa from 41 intraspecies comparisons (Table S3).

Given these results, we selected the ANI-equivalent CGASI value of ≥96.8% to further analyze these taxa. Overall, there were 41 pairwise comparisons of organisms across diverse taxa, including *Arcobacter*, *Caulobacter*, *Neisseria*, *Orientia*, *Ralstonia*, and *Thermus*, that are classically defined as the same species but would be classified as distinct species when assessed using our suggested CGASI threshold (Table S3). Additionally, there were 782 pairwise comparisons of organisms classically defined as different species that this threshold suggests should be the same species, of which only 10 were not in the genus *Rickettsia*, instead belonging to the *Arcobacter* and *Caulobacter* genera (Table S3).

### *Rickettsiaceae* phylogenomic analyses.

**(i) *Rickettsia*.** The *Rickettsiaceae* family includes two genera, the *Rickettsia* and the *Orientia*, and while both genera are obligate intracellular bacteria, *Rickettsia* genomes have undergone more reductive evolution, having a genome size ranging from 1.1 to 1.5 Mbp (48) compared to the 2.0- to 2.2-Mbp size of the *Orientia* genomes (49). Of the *Rickettsiales*, the *Rickettsia* genus contains the greatest number of sequenced genomes and named species, containing 69 genome assemblies in ≤100 contigs representing 27 unique species. *Rickettsia* genomes are currently classified based on the Fournier criteria, an MLST approach established in 2003 based on the sequence similarity of five conserved genes: the 16S rRNA gene, citrate synthase gene (*gltA*), and three surface-exposed protein antigen genes (*ompA*, *ompB*, and gene D) (50). To be considered a *Rickettsia* species, an isolate must have a sequence identity of ≥98.1% to the 16S rRNA and ≥86.5% to *gltA* in at least one preexisting *Rickettsia* species. Within the *Rickettsia*, using *ompA*, *ompB*, and gene D sequence identities, the Fournier criteria also support the further classification of *Rickettsia* species into three groups: the typhus group, the spotted fever group (SFG), and the ancestral group (50). However, the Fournier criteria have not yet been amended to classify the more recently established transitional *Rickettsia* group (51), indicating a need to update the *Rickettsia* taxonomic scheme.

**FIG 3:**
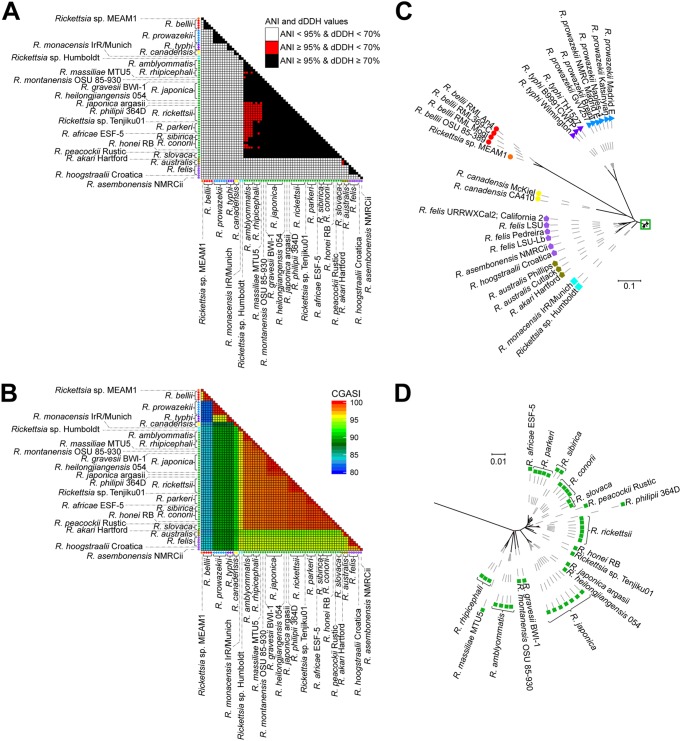
Analysis of the ANI, dDDH, and CGASI values of 69 *Rickettsia* genomes. Across 69 *Rickettsia* genomes, the ANI and dDDH values (A) and CGASI values (B) were calculated for each pairwise genome comparison. The shape next to each *Rickettsia* genome represents whether the genome originates from an ancestral (⚫), transitional (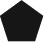), typhus group (▲), or spotted fever group (■) *Rickettsia* species, while the colors of the shapes on the axes represent species designations as determined by a CGASI cutoff of ≥96.8%. (C) An ML phylogenomic tree with 1,000 bootstraps was generated using the core genome alignment. (D) The relationships in the green box in panel C cannot be adequately visualized at the necessary scale, so they are illustrated separately with a different scale. For both trees, red branches represent branches with <100 bootstrap support.

A total of 69 *Rickettsia* genomes representative of 27 different established species were used for ANI, dDDH, and core genome alignment analyses. Regardless of the method used, a major reclassification is justified (Fig. 3; Table S2). A core genome alignment constructed using the 69 *Rickettsia* species genomes yielded a core genome alignment size of ∼0.56 Mbp, 42.4% of the average lengths of the input *Rickettsia* genomes (Table 1). For 42 of the 44 SFG *Rickettsia* genomes, the CGASI between any two genomes is ≥98.2%, well within the proposed CGASI species cutoff of ≥96.8% (Fig. 3), while the CGASI is ≤97.2% in the ancestral and typhus groups. If a CGASI cutoff of 96.8% is used to reclassify the *Rickettsia* species, all but two of the SFG *Rickettsia* genomes would be classified as the same species (Fig. 3), with the two remaining SFG *Rickettsia* genomes, *Rickettsia monacensis* IrR Munich and *Rickettsia* sp. strain Humboldt, being designated as the same species. This is consistent with ANI results as well, while dDDH yields conflicting results (Fig. 3). For the transitional group *Rickettsia*, *Rickettsia akari* and *Rickettsia australis* would be collapsed into a single species due to having CGASI values of 97.2% in a comparison with one another. Similarly, *Rickettsia asembonensis*, *Rickettsia felis*, and *Rickettsia hoogstraalii*, all classified as transitional group *Rickettsia*, would be collapsed into another species, all having CGASI values of 97.2% in comparisons with one another. This is consistent with a phylogenomic tree generated from the *Rickettsia* core genome alignments, where the SFG *Rickettsia* genomes have far less sequence divergence than the rest of the *Rickettsia* genomes (Fig. 3).

### (ii) *Orientia*.

The organisms within *Orientia* have no standardized criteria to define novel species. There are far fewer high-quality *Orientia* genomes than *Rickettsia* genomes, which is due partly to the large number of repeat elements found in *Orientia* genomes, with the genome of Orientia tsutsugamushi being the most highly repetitive sequenced bacterial genome to date, with ∼42% of its genome being comprised of short repetitive sequences and transposable elements (52).

The *Orientia* core genome alignment was constructed using three O. tsutsugamushi genomes and is 0.97 Mbp in size, ∼47.6% of the average input genome size, with CGASI values ranging from 96.3 to 97.2% (Fig. S3). Reclassifying the *Orientia* genomes using a CGASI species cutoff of 96.8% would result in O. tsutsugamushi Gillliam and *O. tsutsugamushi* Ikeda being classified as species separate from O. tsutsugamushi Boryong (Fig. S3). In this case, this reclassification would not be consistent with recommendations from using ANI or dDDH. We suspect that ANI is strongly influenced by the large number of repeats in the genome due to ANI calculations being based off the sequence identity of 1-kbp query genome fragments. In comparison, we do not anticipate that whole-genome alignments would be confounded by the repeats. While the LCBs may be fragmented by the repeats, creating smaller syntenic blocks, the nonphylogenetically informative repeats are eliminated from an LCB-based analysis.

10.1128/mSystems.00236-18.3 FIG S3 Analysis of the ANI, dDDH, and CGASI values of three *Orientia* genomes. For 3 Orientia tsutsugamushi genomes, the ANI and dDDH values (A) and CGASI values (B) were calculated for each genome comparison and color-coded to illustrate the results with respect to cutoffs of an ANI of ≥95% and a dDDH value of ≥70%. Circles of the same colors next to the names of each genome indicate members of the same species as defined by a CGASI of ≥96.8%. Download FIG S3, TIF file, 2.0 MB. Copyright © 2018 Chung et al. 2018 Chung et al. This content is distributed under the terms of the Creative Commons Attribution 4.0 International license.

### *Anaplasmataceae* phylogenomic analyses.

**(i) *Ehrlichia*.** Within the *Anaplasmataceae*, species designations are frequently assigned based on sequence similarity and clustering patterns from phylogenetic analyses generated using the sequences of genes such as 16S rRNA, *groEL*, and *glt*A (53). For example, the species designations for *Ehrlichia khabarensis* and *Ehrlichia ornithorhynchi* are justified based on having a lower sequence similarity for 16S rRNA, *groEL*, and *gltA* (53–55).

**FIG 4:**
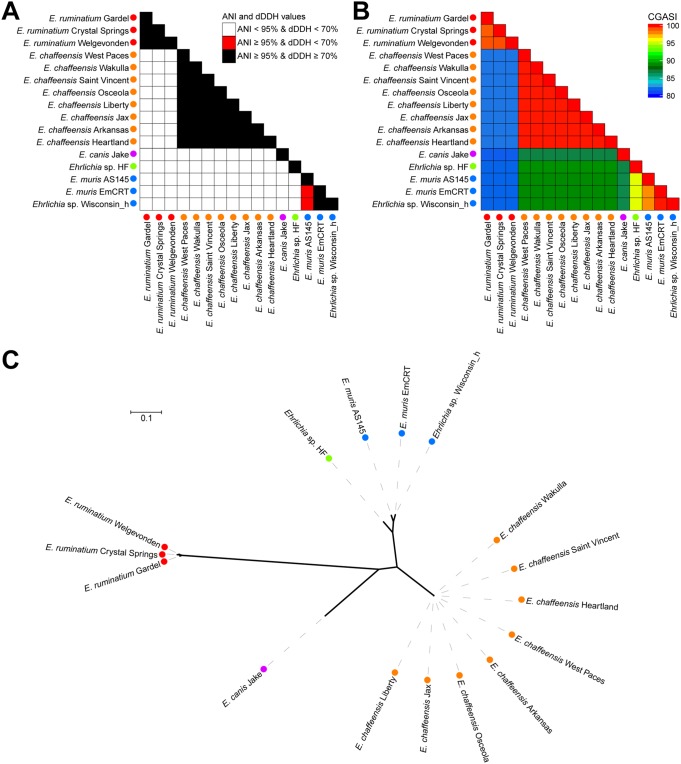
Analysis of the ANI, dDDH, and CGASI values of 16 *Ehrlichia* genomes. For 16 *Ehrlichia* genomes, the ANI and dDDH values (A) and CGASI values (B) were calculated for each pairwise genome comparison. The colors of the shapes next to each *Ehrlichia* genome represent species designations as determined by a CGASI cutoff of ≥96.8%. (C) An ML phylogenomic tree with 1,000 bootstraps was generated using the core genome, with red branches representing branches with <100 bootstrap support.

A core genome alignment constructed using 16 *Ehrlichia* genomes, representative of four defined species, yields a 0.49-Mbp alignment, which equates to 39.8% of the average *Ehrlichia* genome size (1.25 Mbp) (Table 1). Using a CGASI species cutoff of 96.8%, the Ehrlichia chaffeensis and *Ehrlichia ruminantium* genomes were recovered as monophyletic and well-supported species, which is consistent with ANI and dDDH results (Fig. 4). The two Ehrlichia muris and *Ehrlichia* sp. strain Wisconsin_h genomes have CGASI values of >97.8%, indicating that the three genomes represent one species, which is consistent with ANI but not dDDH results (Fig. 4). The genomes of *Ehrlichia* sp. strain HF and *E. canis* Jake do not have CGASI values of ≥96.8% with any other species, confirming their status as individual species, identical to the results found with ANI and dDDH (Fig. 4).

### (ii) *Anaplasma*.

A novel *Anaplasma* species is currently defined based on phylogenetic analyses involving 16S rRNA, *gltA*, and *groEL*, with a new species having a lower sequence identity and a divergent phylogenetic position relative to established *Anaplasma* species (56–59). A core genome alignment constructed using 30 *Anaplasma* genomes yielded a 20-kbp alignment, 1.4% of the average input genome size. As noted before, such low values are indicative of more than one genus being represented in the taxa that were included in the analysis. Thus, CGASI analyses for *Anaplasma* species were done on two *Anaplasma* genome subsets, one containing the 20 Anaplasma phagocytophilum genomes and the other containing 9 Anaplasma marginale genomes and 1 *Anaplasma centrale* genome (Table 1).

**FIG 5:**
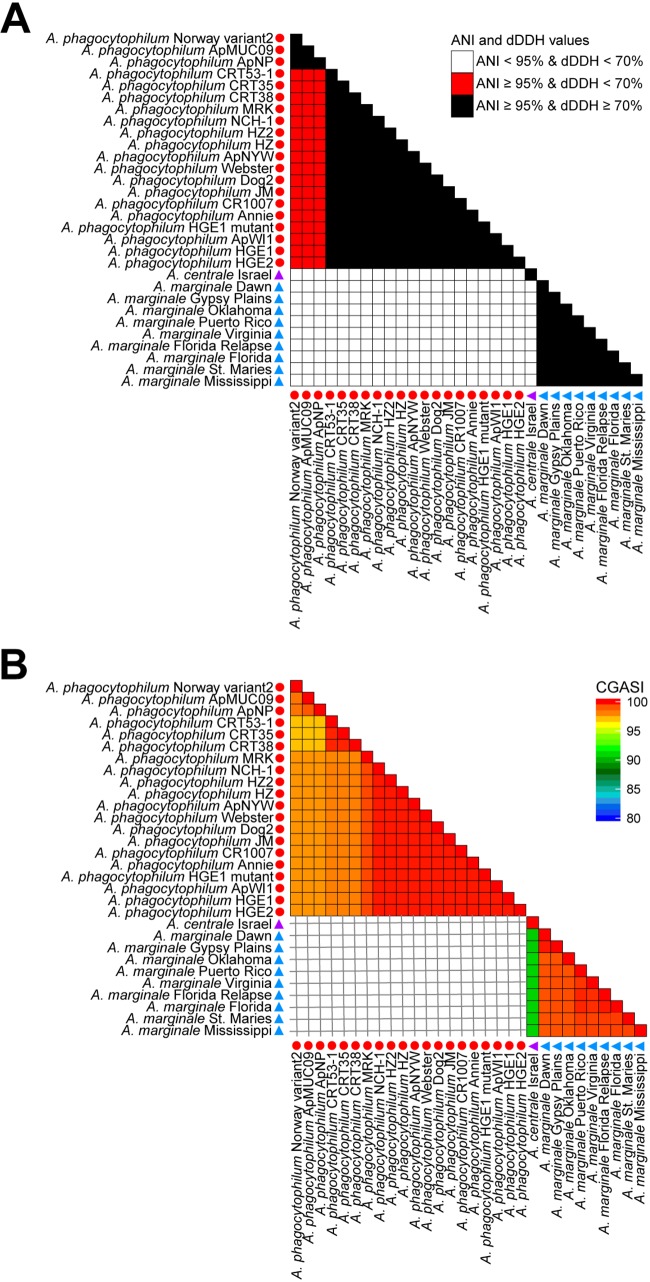
Analysis of the ANI, dDDH, and CGASI values of 30 *Anaplasma* genomes. (A) For 30 *Anaplasma* genomes, the ANI and dDDH values were calculated for each genome comparison and color-coded to illustrate the results with respect to ANI cutoffs of ≥95% and dDDH cutoffs of ≥70%. (B) When we attempted to construct a core genome alignment using all 30 *Anaplasma* genomes, only a 20-kbp alignment was generated, accounting for <1% of the average *Anaplasma* genome size. Therefore, CGASI values were calculated after the *Anaplasma* genomes were split into two subsets containing 20 A. phagocytophilum genomes and the remaining 10 *Anaplasma* genomes. In all panels, the shape next to each genome denotes genus designations as determined by the size of their core genome alignments, while the color of the shape denotes the species as defined by a CGASI cutoff of ≥96.8%.

A 1.25-Mbp core genome alignment, consisting of 39.8% of the average input genome size, was constructed using the 20 A. phagocytophilum genomes. All 20 genomes have CGASI values of ≥96.8%, supporting their designation as members of a single species (Fig. 5). This is supported by ANI, but dDDH again yields conflicting results (Fig. 5). The core genome alignment generated using the remaining 10 *Anaplasma* genomes yields a 0.77-Mbp core genome alignment, 65.3% of the average input genome size. While the A. marginale genomes all have CGASI values of ≥96.8% in comparisons with one another, the Anaplasma centrale genome has CGASI values ranging from 90.7 to 91.0% compared to the nine A. marginale genomes, supporting A. centrale as a separate species from A. marginale, consistent with existing taxonomy and with the ANI and dDDH species cutoffs (Fig. 5).

### (iii) *Neorickettsia*.

The *Neorickettsia* genus contains four genome assemblies: Neorickettsia helminthoeca Oregon, Neorickettsia risticii Illinois, Neorickettsia sennetsu Miyayama, and *Neorickettsia* sp. strain 179522. The genus was first established in 1954 with the discovery of *N. helminthoeca* (60). In 2001, *N. risticii* and *N. sennetsu*, both initially classified as *Ehrlichia* strains, were added to the *Neorickettsia* based on phylogenetic analyses of 16S rRNA and *groESL* (31). A core genome alignment constructed using all four *Neorickettsia* genomes yields a 20-kbp alignment, 2.3% of the average input genome size. When excluding the genome of *N. helminthoeca* Oregon, the three remaining *Neorickettsia* genomes form a core genome alignment of 0.76 Mbp in size, 87.4% of the average input genome size. The sequence identity of the core genome alignment indicates that *N. risticii* Illinois, *N. sennetsu* Miyayama, and *Neorickettsia* sp. 179522 are three distinct species within the same genus, while the length of the core genome alignment indicates that *N. helminthoeca* Oregon is of a separate genus (Fig. S4). When assessing the *Neorickettsia* species designations using ANI and dDDH cutoffs, the four *Neorickettsia* genomes can only be determined to be different species, as the two techniques are unable to delineate phylogenomic relationships at the genus level.

10.1128/mSystems.00236-18.4 FIG S4 Analysis of the ANI, dDDH, and CGASI values of 4 *Neorickettsia* genomes. (A) For the 4 *Neorickettsia* genomes, the ANI and dDDH values were calculated for each genome comparison and color-coded to illustrate the results with respect to cutoffs of an ANI of ≥95% and a dDDH value of ≥70%. (B) CGASI values were calculated and are illustrated using a core genome alignment that could only be constructed using 3 of the *Neorickettsia* genomes, excluding *N. helminthoeca* Oregon. Circles of the sample colors next to the names of each genome indicate members of the same species as defined by a CGASI of ≥96.8%. Download FIG S4, TIF file, 1.9 MB. Copyright © 2018 Chung et al. 2018 Chung et al. This content is distributed under the terms of the Creative Commons Attribution 4.0 International license.

### (iv) *Wolbachia*.

The current *Wolbachia* classification system lacks traditional species designations and instead groups organisms by supergroup designations using an MLST system consisting of 450- to 500-bp internal fragments of five genes: *gatB*, *coxA*, *hcpA*, *ftsZ*, and *fbpA* (61). A core genome alignment generated using 23 *Wolbachia* genomes yields a 0.18-Mbp alignment, amounting to 14.8% of the average input genome size.

Among filarial *Wolbachia* supergroups C and D, the CGASI cutoff of 96.8% would split each of the traditionally recognized supergroups into two groups each, which is also supported by ANI and dDDH. While *w*Oo and *w*Ov would be the same species, *w*Di Pavia should be considered a different species. Similarly, *w*Bm and *w*Wb would be considered the same species, while *w*Ls should be designated a separate species. The *w*Cle, *w*Fol, and *w*Ppe endosymbionts from supergroups E, F, and L, respectively, would all be considered distinct species using CGASI, ANI, or dDDH.

**FIG 6:**
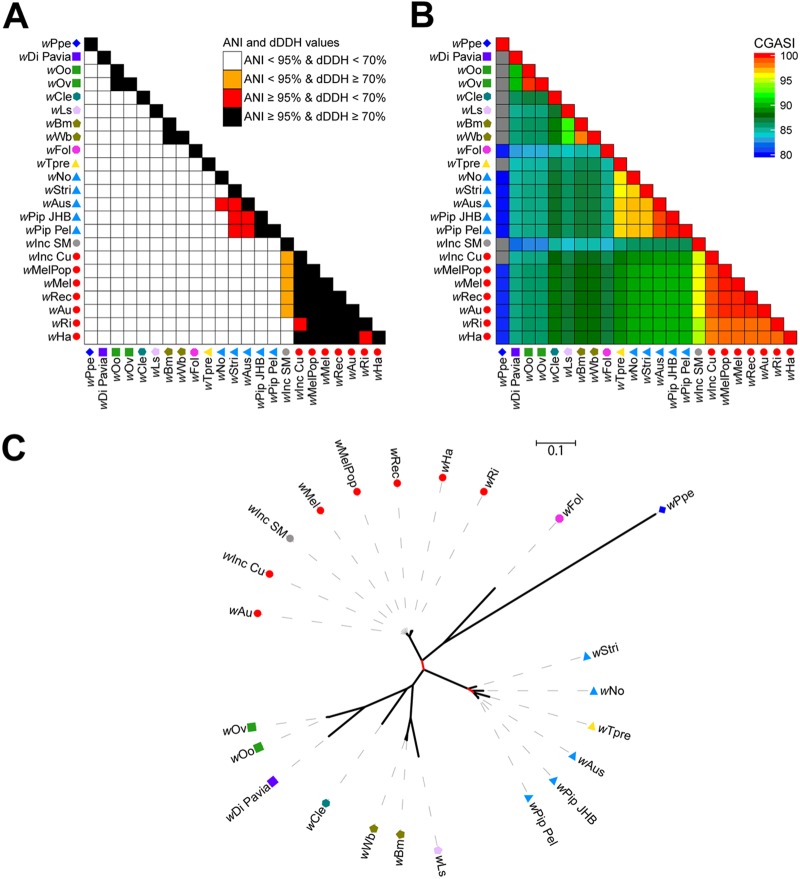
Analysis of the ANI, dDDH, and CGASI values of 23 *Wolbachia* genomes. For 23 *Wolbachia* genomes, the ANI and dDDH values (A) and CGASI values (B) were calculated for each pairwise genome comparison. The shape next to each *Wolbachia* genome represents supergroup A (⚫), B (▲), C (■), D (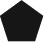), E (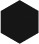), F (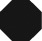), and L (◆) designations, while the color of the shape indicates species as defined by a CGASI cutoff of ≥96.8%. (C) An ML phylogenomic tree was generated using the *Wolbachia* core genome alignment constructed using 23 *Wolbachia* genomes, with red branches representing branches with <100 bootstrap support. The species designations in supergroup B show the CGASI-designated species clusters of *w*Pip_Pel, *w*Pip_JHB, *w*Aus, and *w*Stri to be polyphyletic.

The CGASI values between the supergroup A *Wolbachia* organisms, apart from *w*Inc SM, have CGASI values of ≥96.8 (Fig. 6). The genome of *w*Inc SM has CGASI values ranging from 94.6% to 95.9% compared to other supergroup A *Wolbachia* genomes, indicating that if species assignments were dependent solely on a CGASI cutoff, then *w*Inc SM would be a different species. Because >10% of the positions in the *w*Inc SM genome are ambiguous nucleotide positions, this leads to a large penalty in sequence identity scores, as seen with CGASI, ANI, and DDH (Fig. 6). However, a phylogenomic tree generated using the *Wolbachia* core genome alignment indicates that *w*Inc SM is nested within the other supergroup A *Wolbachia* taxa, a clade with 100% bootstrap support (Fig. 6). Similarly, the results between CGASI, ANI, and dDDH in supergroup B are discordant. Five of the six supergroup B *Wolbachia* genomes, all except for *w*Tpre, have CGASI values of ≥96.8% compared to one of the other five, indicating the five genomes are likely of the same species. However, despite *w*Tpre being considered a different species if the CGASI cutoff of 96.8% is used, a phylogenomic tree constructed using the core genome alignment shows *w*Tpre to be nested within the supergroup B *Wolbachia* organisms (Fig. 6).

In both cases, if *w*Inc SM or *w*Tpre were designated distinct species from the other supergroup A and B *Wolbachia*, respectively, paraphyletic clades would be created. This indicates the importance of using a phylogenomic analysis in addition to a threshold. In this case, the core genome alignment can easily be used with robust phylogenetic algorithms to generate both network trees and ML trees. For both cases, despite *w*Inc SM and *w*Tpre having <96.8% CGASI, the phylogenies reveal that these organisms should be considered the same species as the other supergroup A and B *Wolbachia* species, respectively.

### Other taxa.

The power of an approach that combines phylogenetics with a nucleotide identity threshold is similarly observed with *Neisseria*. A 0.33-Mbp core genome alignment was generated from 66 *Neisseria* genomes, accounting for 14.7% of the average input genome size. The core genome alignment largely supports the classically defined species, including Neisseria meningitidis, Neisseria gonorrhoeae, Neisseria weaveri, and Neisseria lactamica, although one *N. lactamica* isolate, *N. lactamica* 338.rep1_NLAC, appears to be inaccurately assigned (Fig. S5). The CGASI values suggest that each Neisseria elongata isolate is a distinct species, as are the Neisseria flavescens isolates. Like what was observed in *Wolbachia* species, a paraphyletic clade is observed when using a CGASI cutoff of 96.8%, with the genome of *Neisseria* sp. strain HMSC061B04 being nested within a clade of two Neisseria mucosa genomes, *N. lactamica* 338.rep1_NLAC, and several unnamed *Neisseria* taxa while not having a CGASI of ≥96.8% compared to any other *Neisseria* genome (Fig. S5).

10.1128/mSystems.00236-18.5 FIG S5 Analysis of the ANI, dDDH, and CGASI values of 66 *Neisseria* genomes. For the 66 *Neisseria* genomes, the ANI and dDDH values (A) and CGASI values (B) were calculated for each genome comparison and color-coded to illustrate the results with respect to cutoffs of an ANI of ≥95% and a dDDH value of ≥70%. (C) An ML phylogenomic tree was generated using 1,000 bootstraps, with the 0.33-Mbp *Neisseria* core genome alignment. Circles of the sample colors next to the names of each genome indicate members of the same species as defined by a CGASI of ≥96.8. Download FIG S5, TIF file, 2.8 MB. Copyright © 2018 Chung et al. 2018 Chung et al. This content is distributed under the terms of the Creative Commons Attribution 4.0 International license.

## DISCUSSION

### Workflow for the taxonomic assignment of new genomes at the genus and species levels.

These results highlight that while sequence identity cutoffs derived using nucleotide-based pairwise comparisons are important when delineating species, they should be coupled with an analysis of phylogenetic trees. Yet, phylogenetic trees alone are inadequate, as they cannot identify the level of branching where a species should be defined. However, the two results can be quite complementary when applied to core genome alignments and together make for a robust approach.

**FIG 7:**
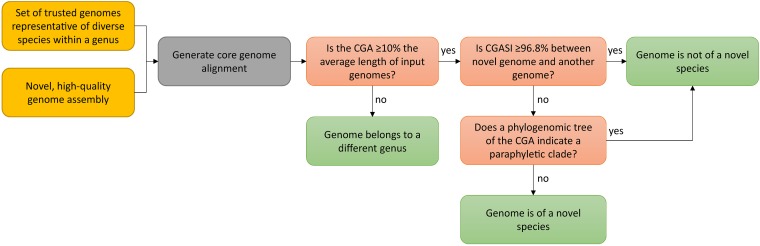
Workflow for the taxonomic assignment of a novel genome at the genus and species levels. The proposed workflow for assigning genus- and species**-**level designations using core genome alignments is based on three criteria: (i) the length of the core genome alignment, (ii) the sequence identity of the core genome alignment, and (iii) phylogenomic analyses. Using a query genome and a set of trusted genomes from a single genus, a core genome alignment is generated. The length of the core genome alignment is the first criterion that is used as the genus-level cutoff, with a core genome alignment size of ≥10% of the average input genome size indicating that all genomes within the subset are of the same genus. Provided that all genomes in the subset are of the same genus, the second criterion is the sequence identity of the core genome alignment, with genomes sharing ≥96.8% similarity being designated the same species. The third and final criterion uses a phylogenomic tree generated using the core genome alignment to check for paraphyletic clades. If the query genome has <96.8% core genome alignment sequence identity with any other genome and its designation as a new species does not form a paraphyletic clade, the genome should be considered a novel species.

Given our results, we propose a workflow using three criteria to guide the taxonomic assignment of novel genomes at the genus and species levels (Fig. 7). Starting with a novel, sequenced query genome, the most closely related genus of the query genome should be identified using homology-based search algorithms (e.g., BLASTN searches of 16S rRNA). Once identified, a set of trusted genomes within the genus should be combined with this query genome to generate a core genome alignment using a tool such as Mauve (27) or Mugsy (28). These trusted genomes would be the type genomes, analogous to type strains. Unlike type strains, they are digitally recorded and thus highly unlikely to be lost. However, multiple organisms with different genome sequences could now serve in the capacity of type strains, eliminating the possibility that a single unusual type strain could unduly influence the taxonomy.

From the core genome alignment, the genus assignment would be considered validated if the length of the core genome alignment is ≥10% of the average input genome size. Subsequently, a pairwise sequence identity matrix is calculated from the core genome alignment, identifying all genomes with ≥96.8% sequence identity. Finally, phylogenomic trees are generated with the core genome alignment and overlaid with the CGASI results. A query genome is deemed a novel species if it is <96.8% identical to the genomes of the type strains and the clades remain monophyletic. Importantly, whole-genome sequences are not needed to assign an existing genus and species designation to a new isolate, merely to describe new genera or species. Community-derived species-specific MLST schemes may be well suited for assigning isolates to already-named and -described species.

### The genomics and data era may necessitate increased flexibility or a modification of the rules in bacterial nomenclature.

The International Code of Nomenclature of Prokaryotes (62) is a frequently updated governance that includes 65 rules that regulate bacterial nomenclature, with the laudable goal of maintaining stability in bacterial names. These rules are designed to assess the correctness of bacterial names, as well as procedures for creating new names, but are agnostic to the methods or criteria used to delineate genera or species (62).

However, modern methods (i.e., genome sequencing, bioinformatics, data sharing, and isolate repositories) have the potential to profoundly and negatively affect bacterial nomenclature in ways where the current code may need to adjust and adapt. Historically, isolates were frequently discovered, described, and named by the same individual or group of individuals. Conflicts arose over time (e.g., two named species turned out to be the same species), and many of the rules focus on resolving those inevitable conflicts. One principle in the code for resolving conflicts relies on using the first published name. However, with modern methods, it would be fairly simple to conduct a comprehensive bioinformatics analysis and be the first to publish names of organisms, without any input from the relevant research communities, including individuals who have spent their scientific careers working on unnamed organisms. In addition, with extensive genomic data, there have been increased efforts to harmonize taxonomy that will lead to large changes in bacterial nomenclature. While choosing the first published name may be effective at resolving small conflicts, it may not be ideal for larger taxonomic changes that may be necessary and warranted. In these cases, the code may need to prioritize large collaborative community-supported efforts that include all relevant stakeholders to reform the nomenclature in a meaningful and important way, particularly efforts that reflect the recent changes in the ways that we think about bacteria and are reflective of the modern conceptual revolutions to which Stephen Jay Gould referred.

Furthermore, a complete genome sequence fully describes an organism, including its phenotypic potential. As such, a genome-informed taxonomic scheme, particularly one that relies on groups of genome type strains, as described here, may render the “*Candidatus*” designation obsolete. “*Candidatus*” was originally proposed as a category or rank given to a potential new taxon that has only sequence data and a morphology determined through microscopy with a nucleic acid probe (63). At that time, this precluded the naming of a species with only a single gene sequence, like 16S rRNA, which could be a chimera. While “*Candidatus*” had an important role at that time and prior to the widespread use of whole-genome sequencing, it has led to extensive confusion among researchers, particularly those new to fields with organisms that use this naming scheme. It also places an undue burden on researchers that study obligate host-associated organisms that are often very highly characterized but unamenable to growth on laboratory media and long-term storage in culture collections. Additionally, among endosymbionts and other obligate intracellular organisms, it has been applied inconsistently, compounding this confusion. Therefore, it seems reasonable that its use should be limited to only organisms from which a complete genome sequence cannot be obtained consistently from the same host organism or host cell line.

### Limitations.

A current issue of whole-genome aligners, such as Mauve or Mugsy, is the inability for the software to scale, being able to computationally handle only subsets of at most ∼80 genomes. While the aligner ParSNP in the Harvest suite can generate a core genome alignment from a larger subset of genomes, its use is limited to intraspecies comparisons (45), making it currently unamenable to this approach. However, for heavily sequenced genera, such as the *Rickettsia* genus, future species assignments for novel genomes do not require constructing a core genome alignment from every available genome. Instead, we recommend that for each genus, the relevant experts in the community establish and curate a set of trusted genomes with at least one representative of each named species that should be used for constructing core genome alignments. After an initial assessment, refinement could be made by using a core genome alignment with many more genomes from closely related species. As an example, in the case of *Rickettsia* species, the initial alignment would include representative genomes from each of the ancestral, typhus, spotted fever, and transitional groups. If the analysis indicated that the query was closest to typhus group genomes, a subsequent alignment would include a curated subset of genomes only from the typhus group. The first core genome alignment would serve to classify a genome into a subgroup, while the second would be used to assign a species designation.

### Conclusions.

As noted with the development of MLST (64), sequence data have the advantage of being incredibly standardized and portable. While MLST methods allow for insight at the subspecies level of taxonomic classifications and are heavily relied upon during infectious disease outbreaks, they do not provide sufficient resolution to define species. Increased resolution is required for taxonomy and can be obtained through the construction of whole-genome alignments that maximize the number of evolutionarily informative positions.

By generating core genome alignments for different genera, we sought to identify and develop a universal, high-resolution method for species classification. Using the core genome alignment, a set of genomes can be assessed based on the length, sequence identity, and a phylogenomic tree of the same core genome alignment. The length of the core genome alignment, which reflects the ability of the genomes to be aligned, is informative in delineating genera. Meanwhile, the pairwise comparisons of core genome alignment sequence identity can be used to delineate species, which can in turn be further refined with a phylogenetic analysis to resolve paraphyletic clades in the taxonomy.

Through this work, we have identified criteria that reconstruct classical species definitions using a method that is transparent and portable. In the course of this work, we have identified modifications that need to be made to the species and genus designations of a number of organisms, particularly within the *Rickettsiales*. While we have identified these instances, we recommend that any changes in nomenclature be addressed by collaborative teams of experts in the respective communities.

## MATERIALS AND METHODS

### Synteny plots.

For the 10 complete *Wolbachia* genomes, NCBI BLAST v2.2.26 with the blastn algorithm was used to obtain the coordinates of the links for each of the pairwise genome comparisons. Synteny plots were generated and visualized using the Artemis Comparative Tool viewer (65, 66).

### Core genome alignments.

The exact commands for generating a core genome alignment from a directory containing all of the fasta files are provided in a bash script (see Text S1 in the supplemental material); the local paths for the bin directors for Mugsy and mothur need to be specified in the script. More specifically, genomes used in the taxonomic analyses were downloaded from NCBI’s GenBank (67). OrthoANIu v1.2 (68) and USEARCH v6.1.544 (69) were run with default settings and used for ANI calculations. GGDC v2.1 (ggdc.dsmz.de) (25) paired with the recommended BLAST+ alignment tool (70) was used to calculate dDDH values. For analyses in this paper, all dDDH calculations were performed using dDDH formula 2 due to the usage of draft genomes in taxonomic analyses (24). For each of the genome subsets, core genome alignments were generated using Mugsy v1.2 (28), with default settings, or Mauve v2.4 (28), with the progressive Mauve algorithm, retaining only LCBs of >30 bp in length that are present in all genomes. mothur v1.22 was used to process each of the core genome alignments to remove positions not present in all organisms in the genome subset (71). Sequence identity matrices for the core genome alignments were created using BioEdit v7.2.5 (brownlab.mbio.ncsu.edu/JWB/papers/1999Hall1.pdf). ML phylogenomic trees with 1,000 bootstraps were calculated for each core genome alignment using IQ-TREE v1.6.2 (72) paired with ModelFinder (73) to select the best model of evolution and UFBoot2 (74) for fast bootstrap approximation. Trees were visualized and annotated using iTOL v4.1.1 (75). Construction of neighbor-network trees was done using the R packages ape (81) and phangorn (77).

10.1128/mSystems.00236-18.6 Text S1 Bash script of commands used to generate a core genome alignment from a directory of whole-genome fasta files. The local paths for Mugsy and mothur must be provided in the bash script. Download Text S1, TXT file, 0.00 MB. Copyright © 2018 Chung et al. 2018 Chung et al. This content is distributed under the terms of the Creative Commons Attribution 4.0 International license.

### Core protein alignments.

Orthologs between complete genomes of the same species were determined using FastOrtho, a reimplementation of OrthoMCL (78) that identifies orthologs using all-by-all BLAST searches, run with default settings. The amino acid sequences of proteins present in all organisms in only one copy were aligned with MAAFT v7.313 FFT-NS-2, using default settings (79). For every protein alignment, the best model of evolution was identified using ModelFinder (73), and phylogenomic trees were constructed using an edge-proportional partition model (80) with IQ-TREE v1.6.2 (72) and UFBoot2 (74) for fast bootstrap approximation. PhyloPhlAn v0.99 was run using default settings as an alternative method of assessing protein-based phylogenies (39). Comparative analyses of core protein and core nucleotide alignment trees were performed using the R packages ape (76) and phangorn (77).

## References

[1] Gould SJ 1983 Hen's teeth and horse's toes. Norton, New York, NY.

[2] Ramasamy D, Mishra AK, Lagier JC, Padhmanabhan R, Rossi M, Sentausa E, Raoult D, Fournier PE 2014 A polyphasic strategy incorporating genomic data for the taxonomic description of novel bacterial species. Int J Syst Evol Microbiol 64:384–391. doi:10.1099/ijs.0.057091-0. 24505076

[3] Schleifer KH 2009 Classification of Bacteria and Archaea: past, present and future. Syst Appl Microbiol 32:533–542. doi:10.1016/j.syapm.2009.09.002. 19819658

[4] Bisen PS, Debnath M, Prasad GBKS 2012 Microbes. Concepts and applications, p 275–338. Wiley Online Library, Hoboken, NJ. doi:10.1002/9781118311912.ch4.

[5] Rossello-Mora R, Amann R 2001 The species concept for prokaryotes. FEMS Microbiol Rev 25:39–67. doi:10.1111/j.1574-6976.2001.tb00571.x. 11152940

[6] Brenner DJ, Fanning GR, Steigerwalt AG 1972 Deoxyribonucleic acid relatedness among species of *Erwinia* and between *Erwinia* species and other enterobacteria. J Bacteriol 110:12–17. 5018020 10.1128/jb.110.1.12-17.1972 PMC247372

[7] Dubnau D, Smith I, Morell P, Marmur J 1965 Gene conservation in *Bacillus* species. I. Conserved genetic and nucleic acid base sequence homologies. Proc Natl Acad Sci U S A 54:491–498. doi:10.1073/pnas.54.2.491. 4956287 PMC219694

[8] Boone DR, Castenholz RW 2001 Bergey's manual of systematic bacteriology: overview: a phylogenetic backbone and taxonomic framework for procaryotic systematics, 2nd ed. Springer, New York, NY.

[9] Quast C, Pruesse E, Yilmaz P, Gerken J, Schweer T, Yarza P, Peplies J, Glockner FO 2013 The SILVA ribosomal RNA gene database project: improved data processing and web-based tools. Nucleic Acids Res 41:D590–D596. doi:10.1093/nar/gks1219. 23193283 PMC3531112

[10] McDonald D, Price MN, Goodrich J, Nawrocki EP, DeSantis TZ, Probst A, Andersen GL, Knight R, Hugenholtz P 2012 An improved Greengenes taxonomy with explicit ranks for ecological and evolutionary analyses of bacteria and archaea. ISME J 6:610–618. doi:10.1038/ismej.2011.139. 22134646 PMC3280142

[11] Palys T, Nakamura LK, Cohan FM 1997 Discovery and classification of ecological diversity in the bacterial world: the role of DNA sequence data. Int J Syst Bacteriol 47:1145–1156. doi:10.1099/00207713-47-4-1145. 9336922

[12] Janda JM, Abbott SL 2007 16S rRNA gene sequencing for bacterial identification in the diagnostic laboratory: pluses, perils, and pitfalls. J Clin Microbiol 45:2761–2764. doi:10.1128/JCM.01228-07. 17626177 PMC2045242

[13] Rosselló-Móra R, Amann R 2015 Past and future species definitions for Bacteria and Archaea. Syst Appl Microbiol 38:209–216. doi:10.1016/j.syapm.2015.02.001. 25747618

[14] Yarza P, Yilmaz P, Pruesse E, Glockner FO, Ludwig W, Schleifer KH, Whitman WB, Euzeby J, Amann R, Rossello MR 2014 Uniting the classification of cultured and uncultured bacteria and archaea using 16S rRNA gene sequences. Nat Rev Microbiol 12:635–645. doi:10.1038/nrmicro3330. 25118885

[15] Chun J, Oren A, Ventosa A, Christensen H, Arahal DR, da Costa MS, Rooney AP, Yi H, Xu XW, De Meyer S, Trujillo ME 2018 Proposed minimal standards for the use of genome data for the taxonomy of prokaryotes. Int J Syst Evol Microbiol 68:461–466. doi:10.1099/ijsem.0.002516. 29292687

[16] Karlsson R, Gonzales-Siles L, Boulund F, Svensson-Stadler L, Skovbjerg S, Karlsson A, Davidson M, Hulth S, Kristiansson E, Moore ER 2015 Proteotyping: proteomic characterization, classification and identification of microorganisms—a prospectus. Syst Appl Microbiol 38:246–257. doi:10.1016/j.syapm.2015.03.006. 25933927

[17] De Bruyne K, Slabbinck B, Waegeman W, Vauterin P, De Baets B, Vandamme P 2011 Bacterial species identification from MALDI-TOF mass spectra through data analysis and machine learning. Syst Appl Microbiol 34:20–29. doi:10.1016/j.syapm.2010.11.003. 21295428

[18] Goris J, Konstantinidis KT, Klappenbach JA, Coenye T, Vandamme P, Tiedje JM 2007 DNA-DNA hybridization values and their relationship to whole-genome sequence similarities. Int J Syst Evol Microbiol 57:81–91. doi:10.1099/ijs.0.64483-0. 17220447

[19] Gevers D, Cohan FM, Lawrence JG, Spratt BG, Coenye T, Feil EJ, Stackebrandt E, Van de Peer Y, Vandamme P, Thompson FL, Swings J 2005 Opinion: re-evaluating prokaryotic species. Nat Rev Microbiol 3:733. doi:10.1038/nrmicro1236. 16138101

[20] Auch AF, von Jan M, Klenk H-P, Göker M 2010 Digital DNA-DNA hybridization for microbial species delineation by means of genome-to-genome sequence comparison. Stand Genomic Sci 2:117–134. doi:10.4056/sigs.531120. 21304684 PMC3035253

[21] Aanensen DM, Spratt BG 2005 The multilocus sequence typing network: mlst.net. Nucleic Acids Res 33:W728–W733. doi:10.1093/nar/gki415. 15980573 PMC1160176

[22] Larsen MV, Cosentino S, Rasmussen S, Friis C, Hasman H, Marvig RL, Jelsbak L, Sicheritz-Ponten T, Ussery DW, Aarestrup FM, Lund O 2012 Multilocus sequence typing of total-genome-sequenced bacteria. J Clin Microbiol 50:1355–1361. doi:10.1128/JCM.06094-11. 22238442 PMC3318499

[23] Kurtz S, Phillippy A, Delcher AL, Smoot M, Shumway M, Antonescu C, Salzberg SL 2004 Versatile and open software for comparing large genomes. Genome Biol 5:R12. doi:10.1186/gb-2004-5-2-r12. 14759262 PMC395750

[24] Auch AF, Klenk HP, Goker M 2010 Standard operating procedure for calculating genome-to-genome distances based on high-scoring segment pairs. Stand Genomic Sci 2:142–148. doi:10.4056/sigs.541628. 21304686 PMC3035261

[25] Meier-Kolthoff JP, Auch AF, Klenk H-P, Göker M 2013 Genome sequence-based species delimitation with confidence intervals and improved distance functions. BMC Bioinformatics 14:60. doi:10.1186/1471-2105-14-60. 23432962 PMC3665452

[26] Tettelin H, Masignani V, Cieslewicz MJ, Donati C, Medini D, Ward NL, Angiuoli SV, Crabtree J, Jones AL, Durkin AS, Deboy RT, Davidsen TM, Mora M, Scarselli M, Margarit y Ros I, Peterson JD, Hauser CR, Sundaram JP, Nelson WC, Madupu R, Brinkac LM, Dodson RJ, Rosovitz MJ, Sullivan SA, Daugherty SC, Haft DH, Selengut J, Gwinn ML, Zhou L, Zafar N, Khouri H, Radune D, Dimitrov G, Watkins K, O'Connor KJ, Smith S, Utterback TR, White O, Rubens CE, Grandi G, Madoff LC, Kasper DL, Telford JL, Wessels MR, Rappuoli R, Fraser CM 2005 Genome analysis of multiple pathogenic isolates of *Streptococcus agalactiae*: implications for the microbial “pan-genome.” Proc Natl Acad Sci U S A 102:13950–13955. doi:10.1073/pnas.0506758102. 16172379 PMC1216834

[27] Darling AC, Mau B, Blattner FR, Perna NT 2004 Mauve: multiple alignment of conserved genomic sequence with rearrangements. Genome Res 14:1394–1403. doi:10.1101/gr.2289704. 15231754 PMC442156

[28] Angiuoli SV, Salzberg SL 2011 Mugsy: fast multiple alignment of closely related whole genomes. Bioinformatics 27:334–342. doi:10.1093/bioinformatics/btq665. 21148543 PMC3031037

[29] Ramírez-Puebla ST, Servín-Garcidueñas LE, Ormeño-Orrillo E, Vera-Ponce de León A, Rosenblueth M, Delaye L, Martínez J, Martínez-Romero E 2015 Species in *Wolbachia*? Proposal for the designation of 'Candidatus *Wolbachia bourtzisii*', 'Candidatus *Wolbachia onchocercicola*', 'Candidatus *Wolbachia blaxteri*', 'Candidatus *Wolbachia brugii*', 'Candidatus *Wolbachia taylori*', 'Candidatus *Wolbachia collembolicola*' and 'Candidatus *Wolbachia multihospitum*' for the different species within *Wolbachia* supergroups. Syst Appl Microbiol 38:390–399. doi:10.1016/j.syapm.2015.05.005. 26189661

[30] Lindsey ARI, Bordenstein SR, Newton ILG, Rasgon JL 2016 Wolbachia pipientis should not be split into multiple species: a response to Ramirez-Puebla et al., “Species in *Wolbachia*? Proposal for the designation of 'Candidatus *Wolbachia bourtzisii*', 'Candidatus *Wolbachia onchocercicola*', 'Candidatus *Wolbachia blaxteri*', 'Candidatus *Wolbachia brugii*', 'Candidatus *Wolbachia taylori*', 'Candidatus *Wolbachia collembolicola*' and 'Candidatus *Wolbachia multihospitum*' for the different species within *Wolbachia* supergroups.” Syst Appl Microbiol 39:220–222. doi:10.1016/j.syapm.2016.03.001. 27021523 PMC5167362

[31] Dumler JS, Barbet AF, Bekker CP, Dasch GA, Palmer GH, Ray SC, Rikihisa Y, Rurangirwa FR 2001 Reorganization of genera in the families Rickettsiaceae and Anaplasmataceae in the order Rickettsiales: unification of some species of Ehrlichia with Anaplasma, Cowdria with Ehrlichia and Ehrlichia with Neorickettsia, descriptions of six new species combinations and designation of Ehrlichia equi and 'HGE agent' as subjective synonyms of Ehrlichia phagocytophila. Int J Syst Evol Microbiol 51:2145–2165. doi:10.1099/00207713-51-6-2145. 11760958

[32] Land M, Hauser L, Jun SR, Nookaew I, Leuze MR, Ahn TH, Karpinets T, Lund O, Kora G, Wassenaar T, Poudel S, Ussery DW 2015 Insights from 20 years of bacterial genome sequencing. Funct Integr Genomics 15:141–161. doi:10.1007/s10142-015-0433-4. 25722247 PMC4361730

[33] Andersson SG, Zomorodipour A, Andersson JO, Sicheritz-Ponten T, Alsmark UC, Podowski RM, Naslund AK, Eriksson AS, Winkler HH, Kurland CG 1998 The genome sequence of *Rickettsia prowazekii* and the origin of mitochondria. Nature 396:133–140. doi:10.1038/24094. 9823893

[34] Ogata H, Audic S, Renesto AP, Fournier PE, Barbe V, Samson D, Roux V, Cossart P, Weissenbach J, Claverie JM, Raoult D 2001 Mechanisms of evolution in *Rickettsia conorii* and *R. prowazekii*. Science 293:2093–2098. doi:10.1126/science.1061471. 11557893

[35] Brayton KA, Kappmeyer LS, Herndon DR, Dark MJ, Tibbals DL, Palmer GH, McGuire TC, Knowles DP. Jr. 2005 Complete genome sequencing of *Anaplasma marginale* reveals that the surface is skewed to two superfamilies of outer membrane proteins. Proc Natl Acad Sci U S A 102:844–849. doi:10.1073/pnas.0406656102. 15618402 PMC545514

[36] Wu M, Sun LV, Vamathevan J, Riegler M, Deboy R, Brownlie JC, McGraw EA, Martin W, Esser C, Ahmadinejad N, Wiegand C, Madupu R, Beanan MJ, Brinkac LM, Daugherty SC, Durkin AS, Kolonay JF, Nelson WC, Mohamoud Y, Lee P, Berry K, Young MB, Utterback T, Weidman J, Nierman WC, Paulsen IT, Nelson KE, Tettelin H, O'Neill SL, Eisen JA 2004 Phylogenomics of the reproductive parasite *Wolbachia pipientis* *w*Mel: a streamlined genome overrun by mobile genetic elements. PLoS Biol 2:E69. doi:10.1371/journal.pbio.0020069. 15024419 PMC368164

[37] Dunning Hotopp JC, Lin M, Madupu R, Crabtree J, Angiuoli SV, Eisen JA, Eisen J, Seshadri R, Ren Q, Wu M, Utterback TR, Smith S, Lewis M, Khouri H, Zhang C, Niu H, Lin Q, Ohashi N, Zhi N, Nelson W, Brinkac LM, Dodson RJ, Rosovitz MJ, Sundaram J, Daugherty SC, Davidsen T, Durkin AS, Gwinn M, Haft DH, Selengut JD, Sullivan SA, Zafar N, Zhou L, Benahmed F, Forberger H, Halpin R, Mulligan S, Robinson J, White O, Rikihisa Y, Tettelin H 2006 Comparative genomics of emerging human ehrlichiosis agents. PLoS Genet 2:e21. doi:10.1371/journal.pgen.0020021. 16482227 PMC1366493

[38] Collins NE, Liebenberg J, de Villiers EP, Brayton KA, Louw E, Pretorius A, Faber FE, van Heerden H, Josemans A, van Kleef M, Steyn HC, van Strijp MF, Zweygarth E, Jongejan F, Maillard JC, Berthier D, Botha M, Joubert F, Corton CH, Thomson NR, Allsopp MT, Allsopp BA 2005 The genome of the heartwater agent *Ehrlichia ruminantium* contains multiple tandem repeats of actively variable copy number. Proc Natl Acad Sci U S A 102:838–843. doi:10.1073/pnas.0406633102. 15637156 PMC545511

[39] Segata N, Bornigen D, Morgan XC, Huttenhower C 2013 PhyloPhlAn is a new method for improved phylogenetic and taxonomic placement of microbes. Nat Commun 4:2304. doi:10.1038/ncomms3304. 23942190 PMC3760377

[40] Parks DH, Chuvochina M, Waite DW, Rinke C, Skarshewski A, Chaumeil PA, Hugenholtz P 2018 A standardized bacterial taxonomy based on genome phylogeny substantially revises the tree of life. Nat Biotechnol 36:996–1004. doi:10.1038/nbt.4229. 30148503

[41] Xia X, Xie Z, Salemi M, Chen L, Wang Y 2003 An index of substitution saturation and its application. Mol Phylogenet Evol 26:1–7. doi:10.1016/S1055-7903(02)00326-3. 12470932

[42] Simmons MP, Ochoterena H, Freudenstein JV 2002 Amino acid vs. nucleotide characters: challenging preconceived notions. Mol Phylogenet Evol 24:78–90. doi:10.1016/S1055-7903(02)00202-6. 12128030

[43] Simmons MP, Carr TG, O'Neill K 2004 Relative character-state space, amount of potential phylogenetic information, and heterogeneity of nucleotide and amino acid characters. Mol Phylogenet Evol 32:913–926. doi:10.1016/j.ympev.2004.04.011. 15288066

[44] Townsend JP, López-Giráldez F, Friedman R 2008 The phylogenetic informativeness of nucleotide and amino acid sequences for reconstructing the vertebrate tree. J Mol Evol 67:437–447. doi:10.1007/s00239-008-9142-0. 18696029

[45] Treangen TJ, Ondov BD, Koren S, Phillippy AM 2014 The Harvest suite for rapid core-genome alignment and visualization of thousands of intraspecific microbial genomes. Genome Biol 15:524. doi:10.1186/s13059-014-0524-x. 25410596 PMC4262987

[46] Philippe H, Forterre P 1999 The rooting of the universal tree of life is not reliable. J Mol Evol 49:509–523. doi:10.1007/PL00006573. 10486008

[47] Walker DH, Ismail N 2008 Emerging and re-emerging rickettsioses: endothelial cell infection and early disease events. Nat Rev Microbiol 6:375–386. doi:10.1038/nrmicro1866. 18414502

[48] Georgiades K, Merhej V, El Karkouri K, Raoult D, Pontarotti P 2011 Gene gain and loss events in *Rickettsia* and *Orientia* species. Biol Direct 6:6. doi:10.1186/1745-6150-6-6. 21303508 PMC3055210

[49] Liao HM, Chao CC, Lei H, Li B, Tsai S, Hung GC, Ching WM, Lo SC 2017 Intraspecies comparative genomics of three strains of *Orientia tsutsugamushi* with different antibiotic sensitivity. Genom Data 12:84–88. doi:10.1016/j.gdata.2017.03.012. 28393016 PMC5377005

[50] Fournier PE, Dumler JS, Greub G, Zhang J, Wu Y, Raoult D 2003 Gene sequence-based criteria for identification of new rickettsia isolates and description of *Rickettsia heilongjiangensis* sp. nov. J Clin Microbiol 41:5456–5465. doi:10.1128/JCM.41.12.5456-5465.2003. 14662925 PMC308961

[51] Gillespie JJ, Beier MS, Rahman MS, Ammerman NC, Shallom JM, Purkayastha A, Sobral BS, Azad AF 2007 Plasmids and rickettsial evolution: insight from *Rickettsia felis*. PLoS One 2:e266. doi:10.1371/journal.pone.0000266. 17342200 PMC1800911

[52] Salje J 2017 *Orientia tsutsugamushi*: a neglected but fascinating obligate intracellular bacterial pathogen. PLoS Pathog 13:e1006657. doi:10.1371/journal.ppat.1006657. 29216334 PMC5720522

[53] Rar VA, Pukhovskaya NM, Ryabchikova EI, Vysochina NP, Bakhmetyeva SV, Zdanovskaia NI, Ivanov LI, Tikunova NV 2015 Molecular-genetic and ultrastructural characteristics of 'Candidatus *Ehrlichia khabarensis*', a new member of the *Ehrlichia* genus. Ticks Tick Borne Dis 6:658–667. doi:10.1016/j.ttbdis.2015.05.012. 26096852

[54] Inokuma H, Brouqui P, Drancourt M, Raoult D 2001 Citrate synthase gene sequence: a new tool for phylogenetic analysis and identification of *Ehrlichia*. J Clin Microbiol 39:3031. doi:10.1128/JCM.39.9.3031-3039.2001. 11526124 PMC88292

[55] Gofton AW, Loh SM, Barbosa AD, Paparini A, Gillett A, Macgregor J, Oskam CL, Ryan UM, Irwin PJ 2018 A novel *Ehrlichia* species in blood and *Ixodes ornithorhynchi* ticks from platypuses (*Ornithorhynchus anatinus*) in Queensland and Tasmania, Australia. Ticks Tick Borne Dis 9:435–442. doi:10.1016/j.ttbdis.2017.12.011. 29284563

[56] Ybanez AP, Matsumoto K, Kishimoto T, Inokuma H 2012 Molecular analyses of a potentially novel *Anaplasma* species closely related to *Anaplasma phagocytophilum* detected in sika deer (*Cervus nippon yesoensis*) in Japan. Vet Microbiol 157:232–236. doi:10.1016/j.vetmic.2011.12.001. 22204789

[57] Li H, Zheng YC, Ma L, Jia N, Jiang BG, Jiang RR, Huo QB, Wang YW, Liu HB, Chu YL, Song YD, Yao NN, Sun T, Zeng FY, Dumler JS, Jiang JF, Cao WC 2015 Human infection with a novel tick-borne *Anaplasma* species in China: a surveillance study. Lancet Infect Dis 15:663–670. doi:10.1016/S1473-3099(15)70051-4. 25833289

[58] Yang J, Liu Z, Niu Q, Liu J, Han R, Liu G, Shi Y, Luo J, Yin H 2016 Molecular survey and characterization of a novel *Anaplasma* species closely related to *Anaplasma capra* in ticks, northwestern China. Parasit Vectors 9:603. doi:10.1186/s13071-016-1886-6. 27884197 PMC5123347

[59] Battilani M, De Arcangeli S, Balboni A, Dondi F 2017 Genetic diversity and molecular epidemiology of *Anaplasma*. Infect Genet Evol 49:195–211. doi:10.1016/j.meegid.2017.01.021. 28122249

[60] Philip CB, Hadlow WJ, Hughes LE 1954 Studies on salmon poisoning disease of canines. I. The rickettsial relationships and pathogenicity of *Neorickettsia helmintheca*. Exp Parasitol 3:336–350. doi:10.1016/0014-4894(54)90032-6. 13183093

[61] Baldo L, Dunning Hotopp JC, Jolley KA, Bordenstein SR, Biber SA, Choudhury RR, Hayashi C, Maiden MC, Tettelin H, Werren JH 2006 Multilocus sequence typing system for the endosymbiont *Wolbachia pipientis*. Appl Environ Microbiol 72:7098–7110. doi:10.1128/AEM.00731-06. 16936055 PMC1636189

[62] Parker CT, Tindall BJ, Garrity GM 2015 International Code of Nomenclature of Prokaryotes. Int J Syst Evol Microbiol 68:1825–1829. doi:10.1099/ijsem.0.000778.

[63] Murray RG, Schleifer KH 1994 Taxonomic notes: a proposal for recording the properties of putative taxa of procaryotes. Int J Syst Bacteriol 44:174–176. doi:10.1099/00207713-44-1-174. 8123559

[64] Maiden MC, Bygraves JA, Feil E, Morelli G, Russell JE, Urwin R, Zhang Q, Zhou J, Zurth K, Caugant DA, Feavers IM, Achtman M, Spratt BG 1998 Multilocus sequence typing: a portable approach to the identification of clones within populations of pathogenic microorganisms. Proc Natl Acad Sci U S A 95:3140–3145. doi:10.1073/pnas.95.6.3140. 9501229 PMC19708

[65] Carver T, Berriman M, Tivey A, Patel C, Bohme U, Barrell BG, Parkhill J, Rajandream MA 2008 Artemis and ACT: viewing, annotating and comparing sequences stored in a relational database. Bioinformatics 24:2672–2676. doi:10.1093/bioinformatics/btn529. 18845581 PMC2606163

[66] Carver TJ, Rutherford KM, Berriman M, Rajandream MA, Barrell BG, Parkhill J 2005 ACT: the Artemis Comparison Tool. Bioinformatics 21:3422–3423. doi:10.1093/bioinformatics/bti553. 15976072

[67] Benson DA, Cavanaugh M, Clark K, Karsch-Mizrachi I, Lipman DJ, Ostell J, Sayers EW 2013 GenBank. Nucleic Acids Res 41:D36–D42. doi:10.1093/nar/gks1195. 23193287 PMC3531190

[68] Yoon SH, Ha SM, Lim J, Kwon S, Chun J 2017 A large-scale evaluation of algorithms to calculate average nucleotide identity. Antonie Van Leeuwenhoek 110:1281–1286. doi:10.1007/s10482-017-0844-4. 28204908

[69] Edgar RC 2010 Search and clustering orders of magnitude faster than BLAST. Bioinformatics 26:2460–2461. doi:10.1093/bioinformatics/btq461. 20709691

[70] Camacho C, Coulouris G, Avagyan V, Ma N, Papadopoulos J, Bealer K, Madden TL 2009 BLAST+: architecture and applications. BMC Bioinformatics 10:421. doi:10.1186/1471-2105-10-421. 20003500 PMC2803857

[71] Schloss PD, Westcott SL, Ryabin T, Hall JR, Hartmann M, Hollister EB, Lesniewski RA, Oakley BB, Parks DH, Robinson CJ, Sahl JW, Stres B, Thallinger GG, Van Horn DJ, Weber CF 2009 Introducing mothur: open-source, platform-independent, community-supported software for describing and comparing microbial communities. Appl Environ Microbiol 75:7537–7541. doi:10.1128/AEM.01541-09. 19801464 PMC2786419

[72] Nguyen LT, Schmidt HA, von Haeseler A, Minh BQ 2015 IQ-TREE: a fast and effective stochastic algorithm for estimating maximum-likelihood phylogenies. Mol Biol Evol 32:268–274. doi:10.1093/molbev/msu300. 25371430 PMC4271533

[73] Kalyaanamoorthy S, Minh BQ, Wong TKF, von Haeseler A, Jermiin LS 2017 ModelFinder: fast model selection for accurate phylogenetic estimates. Nat Methods 14:587–589. doi:10.1038/nmeth.4285. 28481363 PMC5453245

[74] Hoang DT, Chernomor O, von Haeseler A, Minh BQ, Vinh LS 2018 UFBoot2: improving the ultrafast bootstrap approximation. Mol Biol Evol 35:518–522. doi:10.1093/molbev/msx281. 29077904 PMC5850222

[75] Letunic I, Bork P 2016 Interactive Tree of Life (iTOL) v3: an online tool for the display and annotation of phylogenetic and other trees. Nucleic Acids Res 44:W242–W245. doi:10.1093/nar/gkw290. 27095192 PMC4987883

[76] Paradis E, Claude J, Strimmer K 2004 APE: Analyses of Phylogenetics and Evolution in R language. Bioinformatics 20:289–290. doi:10.1093/bioinformatics/btg412. 14734327

[77] Schliep KP 2011 phangorn: phylogenetic analysis in R. Bioinformatics 27:592–593. doi:10.1093/bioinformatics/btq706. 21169378 PMC3035803

[78] Li L, Stoeckert CJ Jr, Roos DS 2003 OrthoMCL: identification of ortholog groups for eukaryotic genomes. Genome Res 13:2178–2189. doi:10.1101/gr.1224503. 12952885 PMC403725

[79] Katoh K, Misawa K, Kuma K, Miyata T 2002 MAFFT: a novel method for rapid multiple sequence alignment based on fast Fourier transform. Nucleic Acids Res 30:3059–3066. doi:10.1093/nar/gkf436. 12136088 PMC135756

[80] Chernomor O, von Haeseler A, Minh BQ 2016 Terrace aware data structure for phylogenomic inference from supermatrices. Syst Biol 65:997–1008. doi:10.1093/sysbio/syw037. 27121966 PMC5066062

[81] Bordenstein SR, Paraskevopoulos C, Dunning Hotopp JC, Sapountzis P, Lo N, Bandi C, Tettelin H, Werren JH, Bourtzis K 2009 Parasitism and mutualism in *Wolbachia*: what the phylogenomic trees can and cannot say. Mol Biol Evol 26:231–241. doi:10.1093/molbev/msn243. 18974066 PMC2721558

