# Supplementary figures and images for: Using Core Genome Alignments To Assign Bacterial Species
Source: mSystems. 2018 Dec 4;3(6):e00236-18. doi: 10.1128/mSystems.00236-18 (PMC6280431; doi:10.1128/mSystems.00236-18)

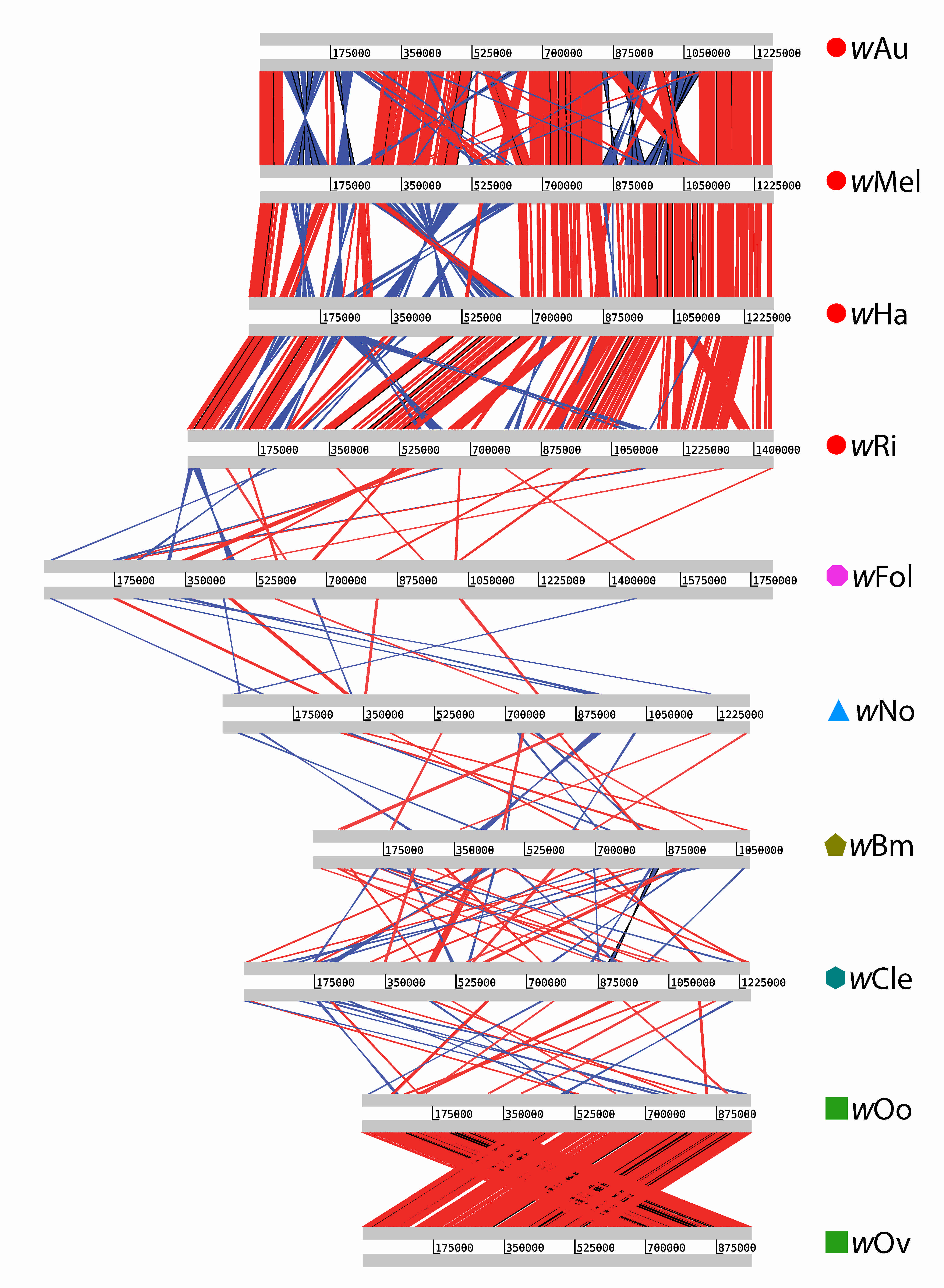

Supplement: FIG S1 [file sys006182290sf1.tif]

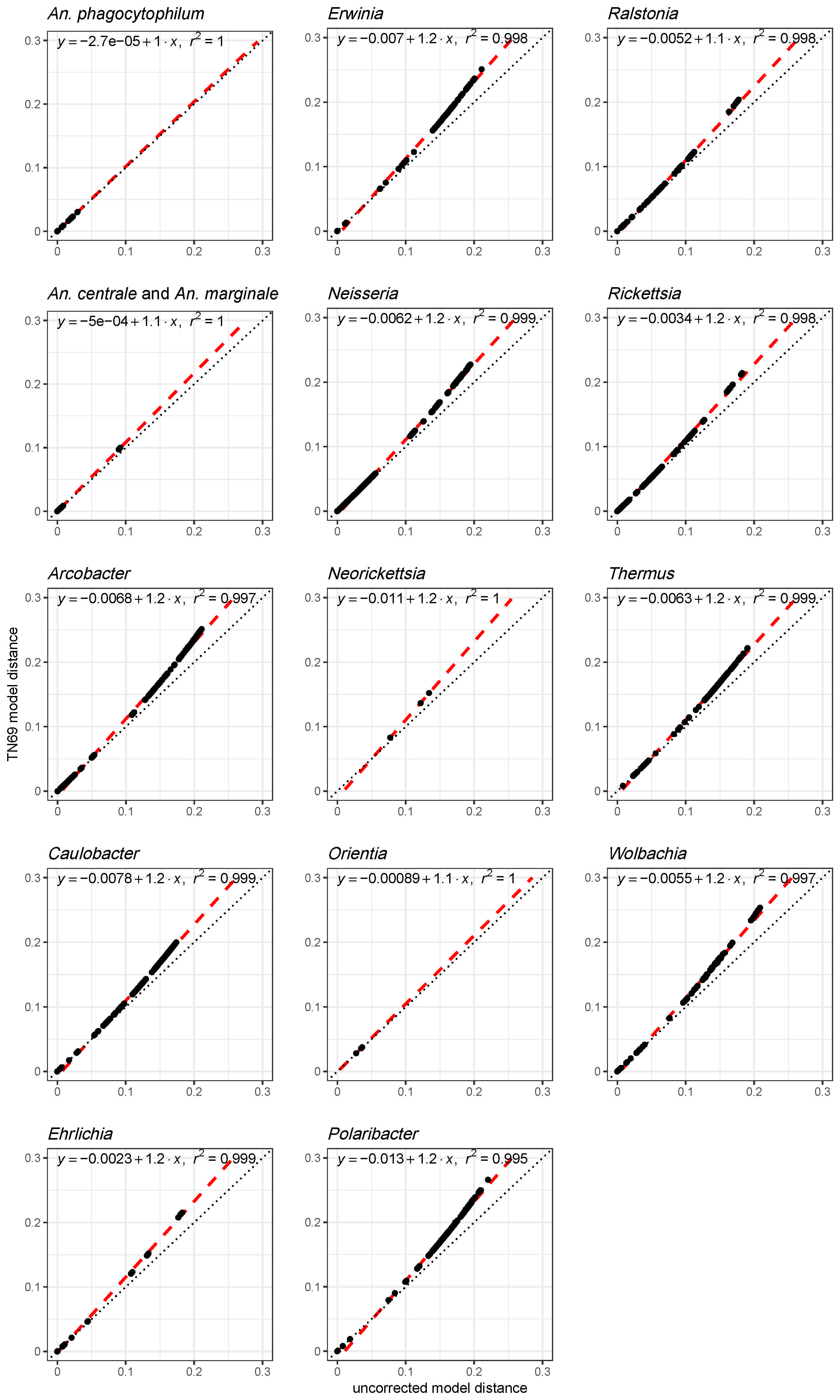

Supplement: FIG S2 [file sys006182290sf2.tif]

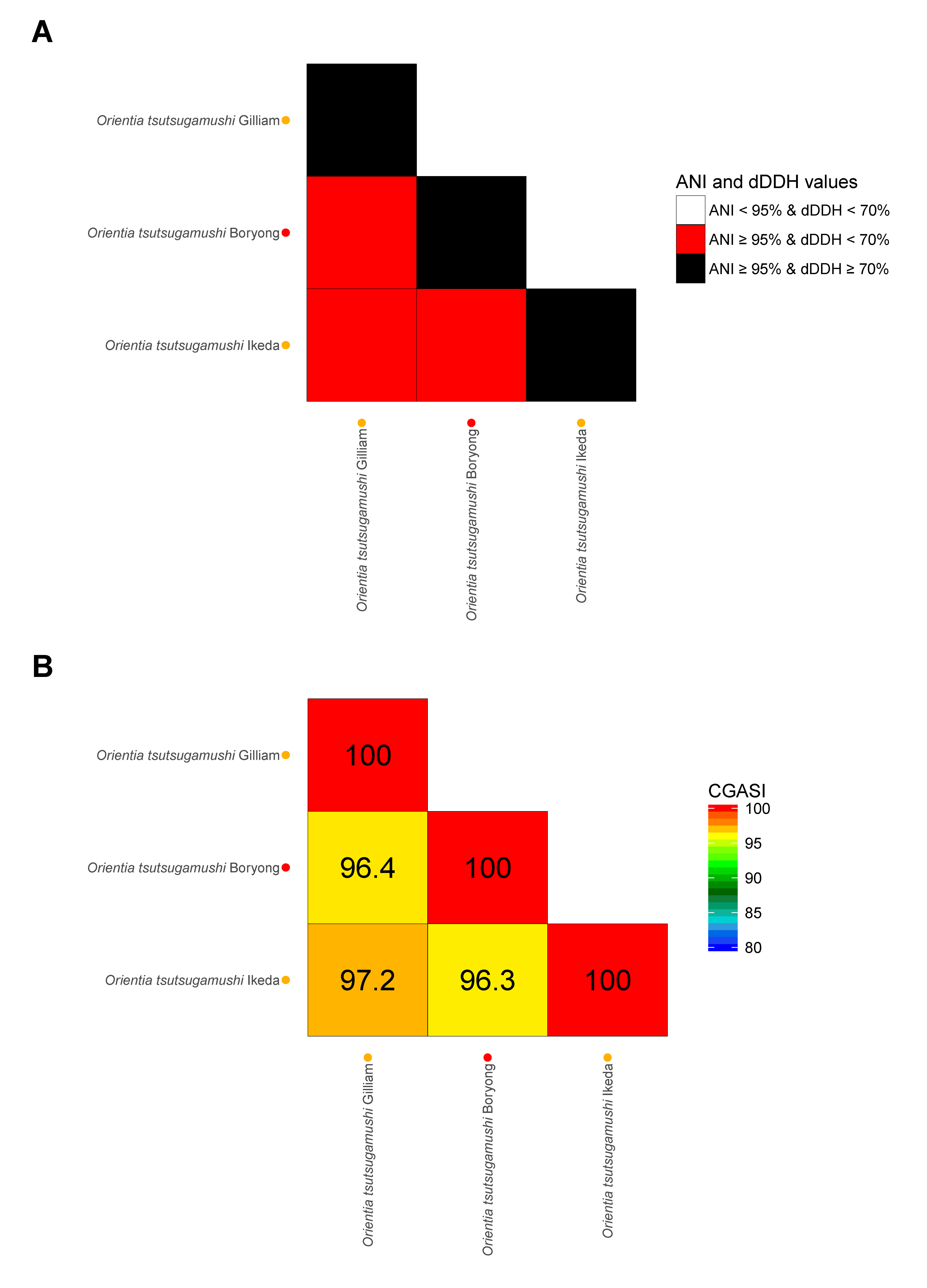

Supplement: FIG S3 [file sys006182290sf3.tif]

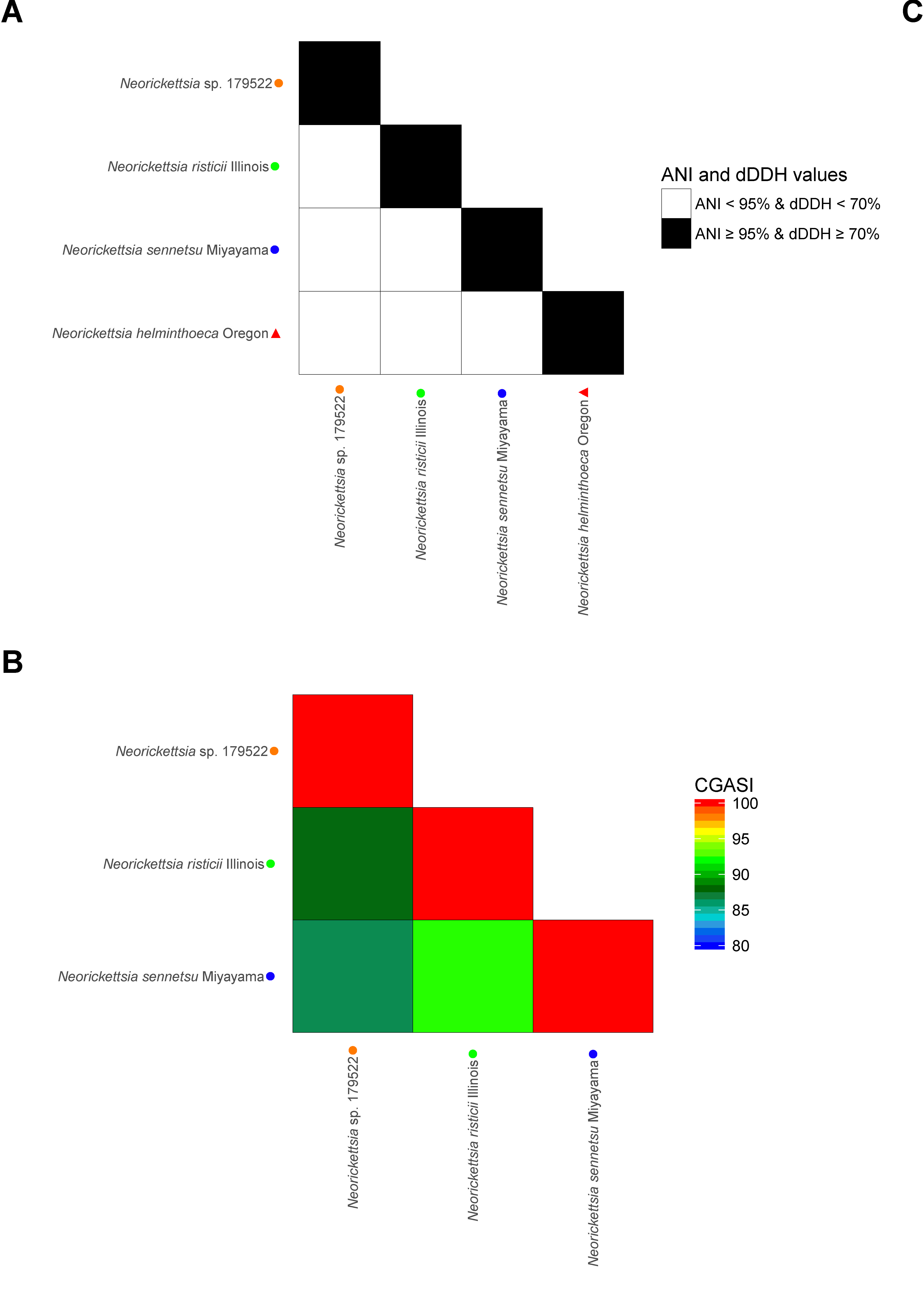

Supplement: FIG S4 [file sys006182290sf4.tif]

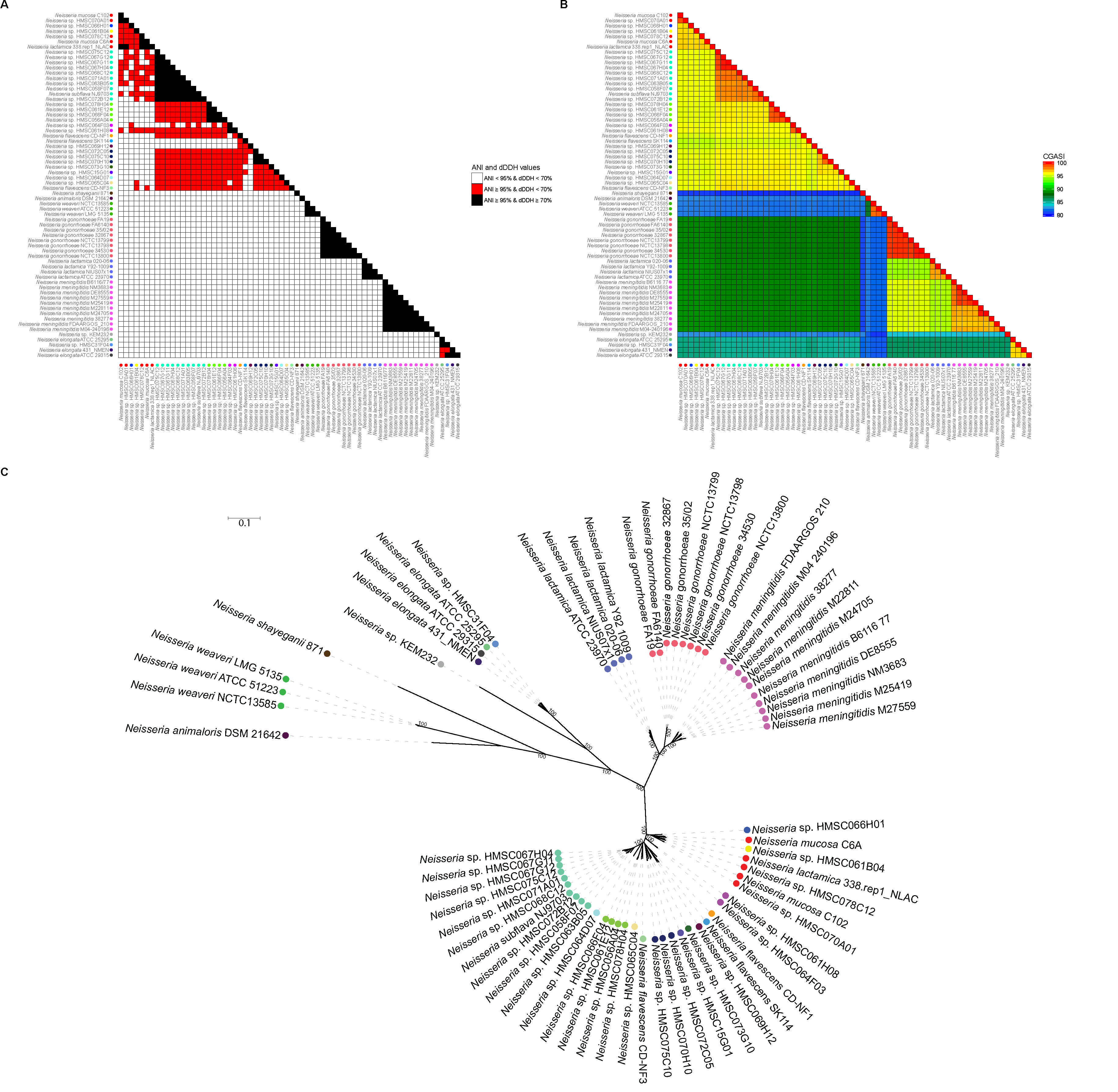

Supplement: FIG S5 [file sys006182290sf5.tif]
